# Two Coupled Low-Barrier Large Amplitude Motions in 3,5-Dimethylanisole Studied by Microwave Spectroscopy

**DOI:** 10.3390/molecules30061195

**Published:** 2025-03-07

**Authors:** Safa Khemissi, Lynn Ferres, Ha Vinh Lam Nguyen

**Affiliations:** 1 Univ Paris Est Créteil and Université Paris Cité, CNRS, LISA, F-94010 Créteil, France; 2 Institute of Physical Chemistry, RWTH Aachen University, Landoltweg 2, D-52074 Aachen, Germany; lynn.ferres@rwth-aachen.de; 3 Institut Universitaire de France (IUF), F-75231 Paris, France

**Keywords:** microwave spectroscopy, large amplitude motions, internal rotation, low barriers

## Abstract

The microwave spectrum of 3,5-dimethylanisole was recorded using a pulsed molecular jet Fourier transform microwave spectrometer, covering the frequency range from 2.0 to 26.5 GHz. Splittings from internal rotations of the *syn*-*m* and *anti*-*m*-methyl groups were observed, analyzed, and modeled using the *XIAM* and the *ntop* programs for a data set including 622 rotational lines. The torsional barriers of the *syn*-*m* and *anti*-*m*-methyl groups were determined to be 58.62367(53) cm^−1^ and 36.28449(69) cm^−1^, respectively. The low barriers to internal rotation of both methyl groups posed significant challenges for spectral analysis and modeling. The successful assignment was achieved using combination difference loops and separately fitting the five torsional components. Comparing the torsional barriers observed in various toluene derivatives with methyl groups at *meta*-positions supports the assumption that electrostatic effects contribute more significantly than steric effects in the low-barrier cases of aromatic molecules.

## 1. Introduction

Rotational spectroscopic investigations into aromatic molecules containing methyl internal rotors are relatively scarce, and studies on those with low torsional barriers are even more limited [1]. This is most likely due to the complexity involved in analyzing and modeling the fine splittings caused by these Large Amplitude Motions (LAMs) [2]. For molecules with one methyl group, all rotational lines split into A-E doublets. The extent of these splittings depends significantly on the methyl torsional barrier. In general, the lower the barrier, the larger the splittings, and the more challenging the spectral analysis and modeling.

Low torsional barriers (say < 100 cm^−1^) often arise from a steric-hindrance-free environment around the methyl rotors, as observed in several monomethyl-substituted aromatic molecules such as *p*-toluic acid (7.9 cm^−1^) [3], *m*-tolunitril (14.2 cm^−1^) [4], *m*-nitrotoluene (6.8 cm^−1^) [5], *p*-tolualdehyde (28.1 cm^−1^) [6], 2-methylthiazole (34.1 cm^−1^) [7], and 3-methylphenylacetylene (11.4 cm^−1^) [8]. The variation in barrier heights is often attributed to electronic effects on the *π*-electron conjugation system caused by other substituents on the aromatic rings. However, a comprehensive understanding of the steric and electronic influences on these barriers remains elusive due to the limited number of studies available.

The complexity increases when dealing with molecules containing two methyl groups, where only a few studies have been reported. In the case of two equivalent methyl groups, the rotational lines split into quartets; with two inequivalent methyl groups, they split into quintets [2]. Most two-top investigations in the literature have focused on molecules with two high barriers, as they are easier to analyze. Molecules with one intermediate barrier and one low barrier, such as *syn*-2,5-dimethylbenzaldehyde [9], 2,5-dimethylfluorobenzene [10], 2,5-dimethylanisole [11], 2,4-dimethylthiazole [12], and 2,3-dimethylanisole [13], are more challenging but have also been studied. Only two toluene derivatives with two low torsional barriers have been investigated: xylene (4.5 cm⁻^1^) [14] and 3,5-dimethylbenzaldehyde (*syn*-*m* 53 cm^−1^ and *anti*-*m* 25.3 cm^−1^) [9]. For these cases, the large splittings between the torsional species significantly complicate the spectral analysis, making accurate modeling of the spectra particularly difficult.

Several program codes can handle two methyl internal rotors. The *XIAM* program [15] is the most widely used in the microwave spectroscopic community. *XIAM* is user-friendly and offers a good balance between calculation accuracy and speed, thanks to efficient matrix factorization and basis transformations. The fitting results are generally satisfactory when the barrier(s) hindering the methyl internal rotation(s) is intermediate (~200–600 cm^−1^) or high (>600 cm^−1^) [16,17,18,19,20,21]. However, *XIAM* often struggles with low barrier cases, failing to achieve standard deviations within the measurement accuracy due to the limited number of available higher-order parameters. Other programs, such as *BELGI-C_s_-2Tops* [22], *PAM-C_2v_-2tops* [23], and *ERHAM* [24], can address this issue more effectively by fitting higher-order parameters [10,25,26]. Another program, *ntop*, also handles low barriers of multiple methyl rotors efficiently and has been successfully applied to two two-top molecules: 2,4-dimethylanisole [27] and 4-methylacetophenone [28,29].

**Figure 1:**
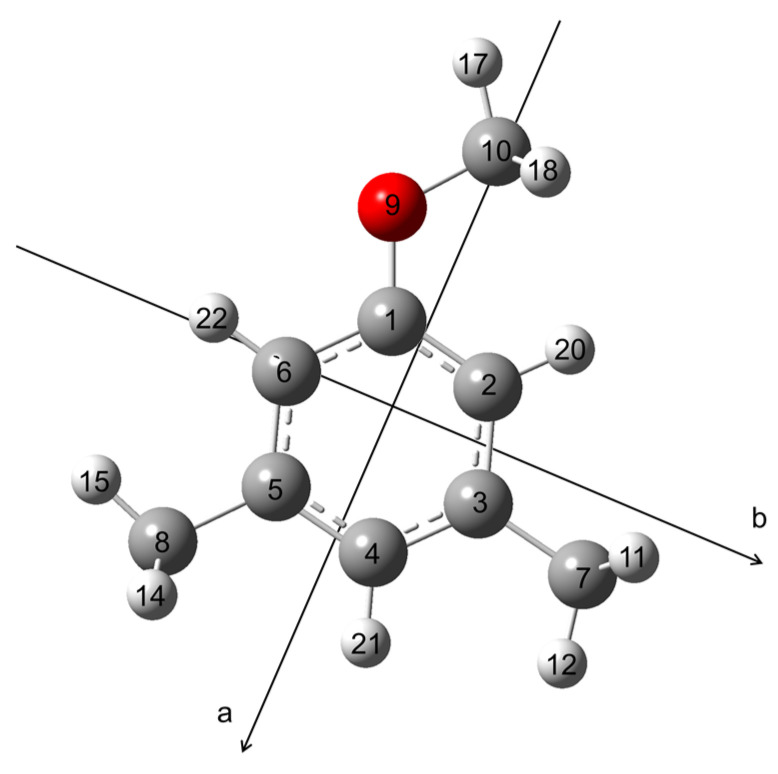
Molecular structure of 35DMA optimized at the B3LYP-D3BJ/6-311++G(d,p) level of theory in the principal axes of inertia. The oxygen atom is red, the carbon atoms are grey, and the hydrogen atoms are white.

In the present study, we report the microwave spectrum of 3,5-dimethylanisole (35DMA, illustrated in Figure 1), which contains two inequivalent methyl groups in the *meta* positions, both undergoing internal rotation hindered by low barriers. We initially applied the *XIAM* program for spectral assignment and fitting, but it failed to achieve a fit with standard deviation within the measurement accuracy. This goal was reached by a subsequent fit with the *ntop* code. We then compared the low torsional barrier values determined for 35DMA with those of the 2,3-, 2,4-, and 3,4-isomers, as well as other toluene derivatives with *meta*-methyl groups, to gain a better understanding of the electronic effects on the methyl torsional barriers.

## 2. Quantum Chemical Calculations

### 2.1. Conformational Analysis

**Figure 2:**
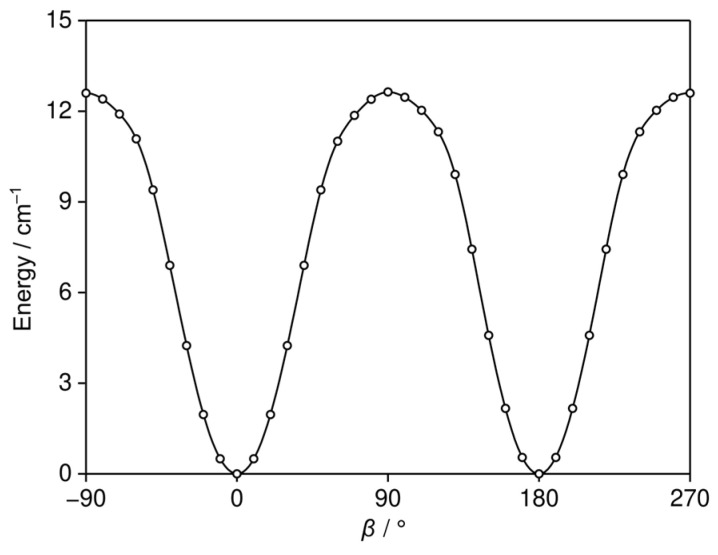
The potential energy curve of 35DMA obtained at the B3LYP-D3BJ/6-311++G(d,p) level of theory by varying the dihedral angle *β* = *∠*(C_2_, C_1_, O_9_, C_10_) in 10° steps, corresponding to the rotation of the methoxy group. The energies are given relative to the lowest value of −425.5592664 Hartree.

All quantum chemical calculations were performed using the *GAUSSIAN 16* software package [30]. To identify the possible conformers of 35DMA, a conformational analysis was conducted by varying the dihedral angle *β* = *∠*(C_2_, C_1_, O_9_, C_10_) in 10° increments, corresponding to a rotation about the C_1_–O_9_ bond (see Figure 1 for atom labeling), while all other geometry parameters were optimized at the B3LYP-D3BJ/6-311++G(d,p) level of theory [31,32,33,34,35], which was used in previous studies on other dimethylanisoles. The potential energy curve is shown in Figure 2. This level of theory predicted two equivalent minima at *β* = 0° and *β* = 180°, corresponding to two identical molecular structures of 35DMA.

### 2.2. Basis Set Variation

Geometry optimizations were carried out at the B3LYP-D3BJ/6-311++G(d,p) [31,32,33,34,35], MP2/6-311++G(d,p) [35,36], and MP2/6-31G(d,p) [35,36] levels of theory. These levels were chosen for their ability to produce reliable rotational constants that closely match experimental values for various six-membered aromatic molecules with methyl groups, such as the five isomers of the dimethylfluorobenzene family [10,37,38,39,40] and the 2,3- [13], 2,4- [27], 3,4-dimethylanisole [41] isomers. Geometry optimizations were followed by harmonic frequency calculations to verify that the obtained structure is a real minimum and anharmonic frequency calculations to access ground state rotational constants and centrifugal distortion constants. The main contribution of the difference between the ground state rotational constants and the equilibrium rotational constants is the vibration–rotation interaction, which is, to a first approximation, one-half the sum of the *α* values from each of the fundamental vibrations. The structure optimized at the B3LYP-D3BJ/6-311++G(d,p) level is illustrated in Figure 1. The calculated equilibrium rotational constants, vibrational ground state rotational constants, centrifugal distortion constants, and dipole moment components are presented in Table 1. The atomic Cartesian coordinates are given in Table S1 in the Supplementary Materials.

For benchmarking purposes, and to offer alternative levels beyond MP2/6-31G(d,p), MP2/6-311++G(d,p), and B3LYP-D3BJ/6-311++G(d,p), geometry optimizations were performed using various methods and basis sets. Calculations employed density functional theory (DFT) methods including B3LYP-D3 [31,32,33], B3LYP-D3BJ [31,32,33,34], CAM-B3LYP-D3BJ [42], M06-2X [43], ωB97X-D [44], MN15 [45], and PBE0 [46]. Additionally, the ab initio MP2 [36] and CCSD [47] methods were used. These methods were combined with a range of Pople [35] and Dunning [48] basis sets, except for the coupled-cluster method, which was combined exclusively with the cc-pVDZ basis set. The predicted rotational constants are listed in Table S2 of the Supplementary Materials.

### 2.3. Methyl Internal Rotations

The molecule 35DMA features a methoxy group and two inequivalent methyl groups undergoing internal rotation, located at the *syn*-*meta* and *anti*-*meta* positions. Previous studies have estimated the barrier hindering the methoxy methyl internal rotation in anisole to be approximately 1200 cm^−1^ [49]. Prior research by Ferres et al. on 2,3- [13], 2,4- [27], and 3,4-dimethylanisole [41] has demonstrated that no splittings resulting from the methoxy methyl torsion are observed in their microwave spectra. For 35DMA, the barrier to internal rotation was calculated at the MP2/6-31G(d,p), MP2/6-311++G(d,p), and B3LYP-D3BJ/6-311++G(d,p) levels of theory by varying the dihedral angle *φ* = *∠*(C_1_, O_9_, C_10_, H_17_) in 10° increments. Due to the three-fold C_3_ symmetry of the methyl group, a rotation of 120° was sufficient. The calculated *V*_3_ potential terms were 1081.8 cm^−1^, 981.0 cm^−1^, and 1070.5 cm^−1^, respectively. These results indicate that no resolvable splittings arising from the internal rotation of the methoxy methyl group are expected to be observed in the microwave spectrum.

**Figure 3:**
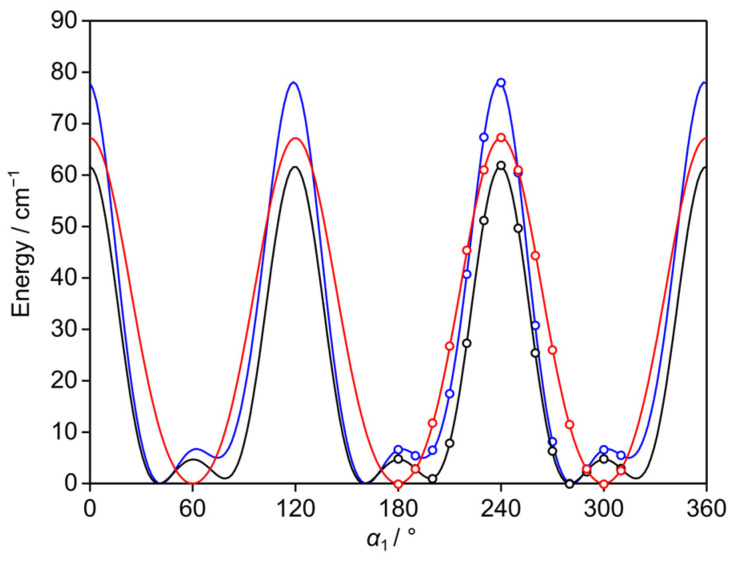
The potential energy curves of 35DMA calculated at the B3LYP-D3BJ/6-311++G(d,p) (red curve), MP2/6-311++G(d,p) (blue curve), and MP2/6-31G(d,p) (black curve) levels of theory by varying the dihedral angle *α*_1_ = *∠*(C_2_, C_3_, C_7_, H_12_) in 10° increments, corresponding to the rotation of the 3-methyl group (*syn*-*m*) about the C_3_–C_7_ bond.

**Figure 4:**
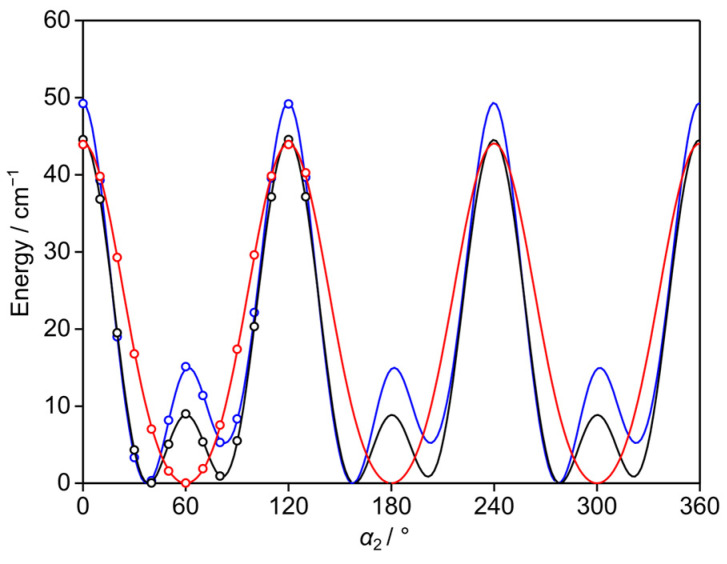
The potential energy curves of 35DMA calculated at the B3LYP-D3BJ/6-311++G(d,p) (red curve), MP2/6-311++G(d,p) (blue curve), and MP2/6-31G(d,p) (black curve) levels of theory by varying the dihedral angle *α*_2_ = *∠*(C_4_, C_5_,C_8_, H_15_) in 10° increments, corresponding to the rotation of the 5-methyl group (*anti*-*m*) about the C_5_–C_8_ bond.

Low barriers hindering the internal rotations of the two inequivalent methyl groups at the 3- and 5-positions (*syn*-*m* and *anti*-*m*, respectively) were anticipated due to their steric-free environment. To calculate the barrier heights, the respective dihedral angles *α*_1_ = *∠*(C_2_, C_3_, C_7_, H_12_) and *α*_2_ = *∠*(C_4_, C_5_, C_8_, H_15_) were also varied in 10° increments, while optimizing all other parameters. The resulting potential energies were parameterized using a 1D-Fourier expansion, with the corresponding coefficients provided in Table S3 of the Supplementary Materials. The potential energy curves are shown in Figure 3 and Figure 4. At the B3LYP-D3BJ/6-311++G(d,p) level of theory, calculations yielded typical three-fold potentials for both the *syn*-*m* and *anti*-*m*-methyl groups, with *V*_3_ terms of 67.2 cm^−1^ and 44.0 cm^−1^, respectively. Small *V*_6_ terms of about 4.8 cm^−1^ and 7.2 cm^−1^ were obtained. At both the MP2/6-311++G(d,p) and MP2/6-31G(d,p) levels, asymmetric potential energy curves were observed, with significant *V*_6_ contributions. For the *syn*-*m*-methyl group, the *V*_3_/*V*_6_ ratios were calculated as 69.2 cm^−1^/29.4 cm^−1^ and 55.2 cm^−1^/26.2 cm^−1^, respectively. For the *anti*-*m*-methyl group, the respective *V*_3_/*V*_6_ ratios are 34.6 cm^−1^/26.2 cm^−1^ and 36.8 cm^−1^/22.0 cm^−1^. The asymmetry observed in the potential energy curves at the MP2 levels suggests a significant interaction between the internal rotors, as further evidenced by the potential energy surfaces, as described in Section 2.4.

### 2.4. Potential Energy Surfaces

**Figure 5:**
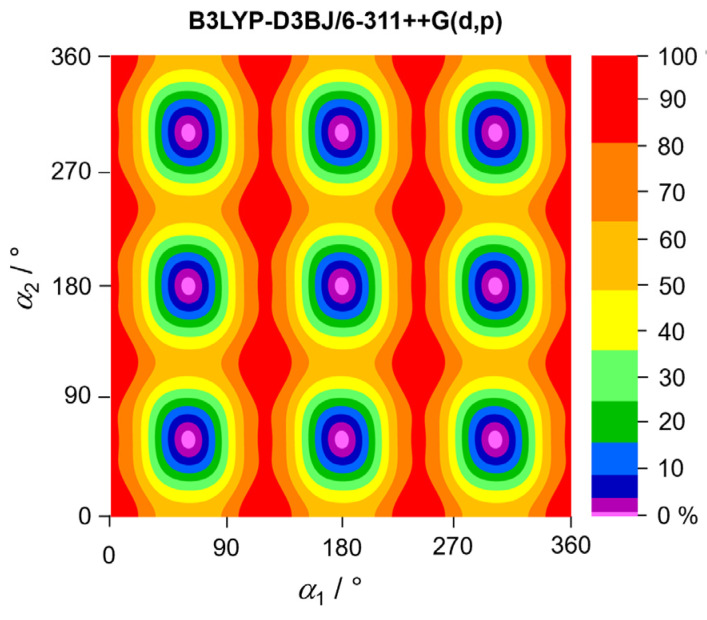
Two-dimensional potential energy surface of 35DMA as a function of the dihedral angles *α*_1_ = *∠*(C_2_, C_3_, C_7_, H_12_) and *α*_2_ = *∠*(C_4_, C_5_, C_8_, H_15_), calculated at the B3LYP-D3BJ/6-311++G(d,p) level of theory. The dihedral angles were varied in 10° increments, while all other geometry parameters were optimized. The numbers in the color code indicate the energy (in percent) relative to the energetic minimum (0%) and maximum (100%), with *E*_min_ = −425.559266 Hartree and *E*_max_ = −425.558897 Hartree. Note that a non-linear scale is used.

**Figure 6:**
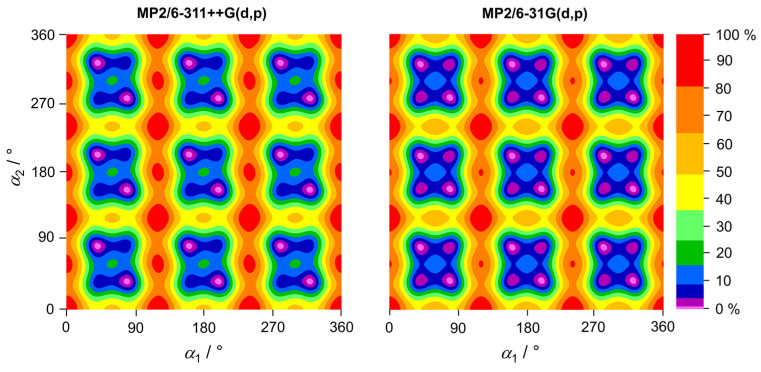
Two-dimensional potential energy surfaces of 35DMA as a function of the dihedral angles *α*_1_ and *α*_2_, calculated at the MP2/6-311++G(d,p) and MP2/6-31G(d,p) levels of theory. The dihedral angles were varied in 10° increments, while all other geometry parameters were optimized. The numbers in the color code indicate the energy (in percent) relative to the energetic minimum (0%) and maximum (100%), with *E*_min_ = −424.244142 Hartree and *E*_max_ = −424.243628 Hartree for the MP2/6-311++G(d,p) level, and *E*_min_ = −424.083418 Hartree and *E*_max_ = −424.082994 Hartree for the MP2/6-31G(d,p) level.

The coupling between the methyl internal rotations in aromatic molecules through π conjugation, particularly when hindered by low barriers, can complicate the spectral assignment and modeling. To gain insight into this coupling, we calculated two-dimensional potential energy surfaces (2D-PES) based on the dihedral angles *α*_1_ and *α*_2_ at the B3LYP-D3BJ/6-311++G(d,p), MP2/6-311++G(d,p), and MP2/6-31G(d,p) levels of theory. Both *α*_1_ and *α*_2_ were varied in 10° increments, while all other geometry parameters were optimized. The resulting potential energy points were parameterized using a 2D-Fourier expansion, with the coefficients provided in Table S4 of the Supplementary Materials. The 2D-PES plots are shown in Figure 5 and Figure 6. The 2D-PES calculated at the B3LYP-D3BJ/6-311++G(d,p) level exhibits minima with an oblate, stretched shape rather than a circular shape, indicating a coupling between the two tops. This coupling can also be recognized by the significant *V_cc_* term cos(3*α*_1_)cos(3*α*_2_), as shown in Table S4 of the Supplementary Materials. The 2D-PES calculated at the MP2/6-311++G(d,p) and MP2/6-31G(d,p) levels revealed the presence of asymmetric double minima, consistent with the 1D potential curves illustrated in Figure 3 and Figure 4 and confirmed the substantial coupling of the two rotors (note also the *V_cc_* terms in Table S4).

## 3. Microwave Spectroscopy

### 3.1. Measurements

**Figure 7:**
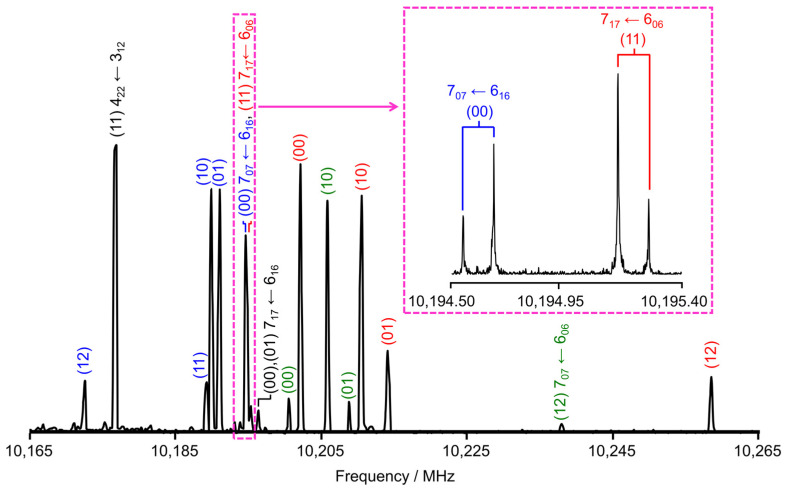
A portion of the survey scan from 10,165 MHz and 10,265 MHz recorded by overlapping spectra with 0.25 MHz step width and 50 co-added FIDs per each single measurement. The absolute intensity is presented in arbitrary units. All lines are labeled with their torsional species (00), (10), (01), (11), and (12) as well as their corresponding rotational quantum numbers $J_{K_{a}^{\prime }K_{c}^{\prime }}^{\prime }\leftarrow J_{K_{a}K_{c}}$. Inset: a measurement at high resolution showing the (00) and (11) torsional species of the 7_07_ ← 6_16_ and 7_17_ ← 6_06_ rotational transitions, respectively. Doppler doublets are marked by brackets.

The microwave spectra were recorded using a pulsed molecular jet Fourier transform microwave spectrometer with a coaxially-oriented beam-resonator arrangement (COBRA) [50] in the frequency range of from 2.0 to 26.5 GHz. The substance, with a purity of 99%, was purchased from Alfa Aesar, Karlsruhe, Germany. A few drops of the sample were placed on a 5 cm piece of pipe cleaner inserted into a stainless steel tube mounted upstream of the nozzle. Helium was passed over the sample at a backing pressure of 2 bar, and the resulting Helium-35DMA mixture was expanded into the cavity. In the frequency range from 9.8 to 13.3 GHz, a survey scan was recorded by overlapping spectra with 50 co-added free induction decays (FIDs) for each spectrum at a step width of 0.25 MHz. All signals observed in the survey scan were remeasured at higher resolution, where each line appeared as doublets due to the Doppler effect caused by the COBRA setup. The measurement accuracy is estimated to be approximately 4 kHz. A scan portion and a typical high-resolution spectrum showing the (00) and (11) torsional species of the 7_07_ ← 6_06_ and 7_17_ ← 6_06_ rotational transitions, respectively, are shown in Figure 7.

### 3.2. Spectral Assignments

As a first step, the assignment was initiated by treating the molecule as a rigid-rotor with no internal rotation effects, focusing only on the (00) torsional species. Using the rotational constants and dipole moment components calculated at the B3LYP-D3BJ/6-311++G(d,p) level of theory (see Table 1), we predicted the theoretical microwave spectrum using the *XIAM* program [15]. This level of theory was chosen because it has predicted rotational constants that closely match experimental values in previous studies on other dimethylanisole isomers [13,27,41], and was also specifically recommended to guide the spectral assignment of similar molecular systems, such as the dimethylfluorobenzene family [10,37,38,39,40]. Given the dipole moment components in Table 1, the spectrum is expected to display strong *b*-type transitions, weaker *a*-type transitions, and an absence of *c*-type transitions. We concentrated first on identifying intense *b*-type transitions with low *J* values (*J*_max_ = 5) and low *K_a_* values (*K_a_* = 0, 1), followed by *a*-type transitions. This assignment process was straightforward. As a next step, we searched for transitions with higher *J* and *K_a_* values, completing the rigid-rotor assignment.

**Figure 8:**
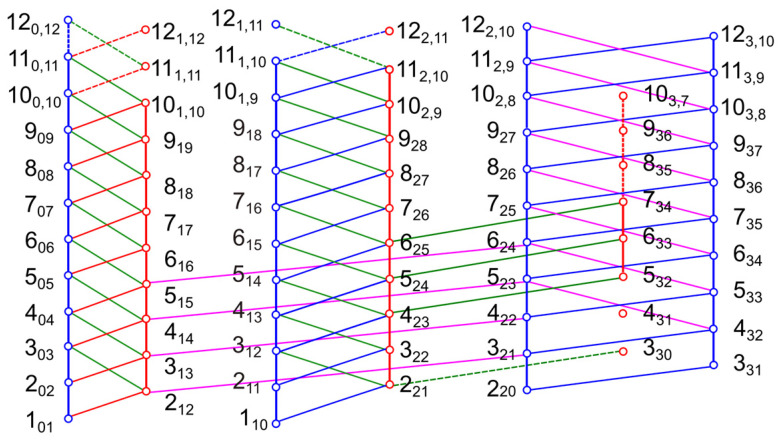
Schematic illustration of almost all observed rotational transitions for the (10), (01), (11), and (12) torsional components of 35DMA. The solid lines connecting the circles represent transitions verified by combination difference loops, which sum to a measurement accuracy of 4 kHz. Some weak transitions with high *J* and *K_a_* could not be measured, and some loops remain open. Transitions involved in such loops are indicated as dashed lines.

Due to the high barrier to internal rotation of the methoxy methyl group, the spectrum is expected to exhibit torsional splittings arising from the internal rotations of only the two inequivalent *syn*-*m* and *anti*-*m*-methyl groups. Each rotational transition splits into five torsional components (00), (10), (01), (11), and (12) [41]. The low barriers to internal rotation of both methyl groups result in large spectral splittings, making the assignment process challenging. To analyze these complex LAMs, we first focused on the (10) species associated with the *syn*-*m*-methyl torsion, given its higher predicted barrier (see Table 1). We calculated a theoretical one-top spectrum using the rotational constants obtained from the rigid-rotor fit as well as the *V*_3,1_ potential term and the angle *∠*(*i*_1_,*a*) from the B3LYP-D3BJ/6-311++G(d,p) calculations. Again, we targeted first the intense *b*-type transitions with low *J* and *K_a_* values, especially the (*J*+1)_0(*J*+1)_ ← *J*_1*J*_ branch, as this branch has the smallest splittings of all *b*-type lines. We then moved on to assign *a*-type transitions, aiming to find closed combination difference loops (Ritz cycles) [51] to verify the accuracy of the assigned lines (see Figure 8). Subsequently, we fitted the loop-checked (10) lines separately using the *SFLAMS* program [28], incorporating odd power parameters in the $H_{op}$ term of the Hamiltonian, as shown in Equation (1):

(1) $$H_{op}=\left(q+q_{J}P^{2}+q_{K}P_{z}^{2}\right)P_{z}+\left(r+r_{J}P^{2}\right)P_{x}+\frac{1}{2}r_{K}\left\{P_{z}^{2},P_{x}\right\}.$$

With predictions from the separate fit, further higher *J* and *K_a_* transitions were identified, extending the loops as well as the *XIAM* and *SFLAMS* fits.

After assigning the (10) torsional species, we repeated the same procedure to assign the (01) species associated with the internal rotation of the *anti*-*m*-methyl group, including checking with Ritz cycles and separate fits. Then, we combined the two *XIAM* one-top fits to a two-top fit to predict and search for the interaction (11) and (12) components. After numerous trials, we successfully assigned a sufficient number of lines which were verified using combination difference loops and separate fits with *SFLAMS*. Predictions from these fits subsequently guided further assignments. Given the C_s_ symmetry of the molecular frame, *c*-type transitions were not expected in the microwave spectrum of 35DMA, as the *c*-component of the dipole moment is zero. However, some *c*- and *x*-type forbidden transitions were observed for the (10), (01), (11), and (12) torsional species. For those perturbation-allowed transitions, the *K_a_* and *K_c_* quantum numbers have lost their meaning and only indicate the energy order. These transitions are highlighted in the frequency list in Table S5 of the Supplementary Materials. The molecular parameters of the five separate fits are given in Table 2.

## 4. Results of the Global Fits and Discussion

In total, 622 torsional lines were assigned. The data set was fitted globally with the *XIAM* program [15], yielding a standard deviation of 347.5 kHz, while the measurement accuracy is 4 kHz. To improve the quality of the global fit, we applied the *ntop* program [27] to the same data set, successfully decreasing the rms deviation to 5.3 kHz by incorporating a larger number of higher-order parameters. The molecular parameters obtained using the *XIAM* and *ntop* codes are presented in Table 3. A list of all fitted frequencies along with their residuals is available in Table S5 in the Supplementary Materials. Due to the strong correlation between the *V*_3_ terms and the internal rotation constants *F*_0_, we fixed *F*_0_ to 160 GHz, a value often found for methyl groups, in both the *XIAM* and *ntop* fits. Note that if we fit *F*_0_ in *XIAM* and include additional high-order parameters (*V_J_*, *V_K_,* and *V_−_*, also referred to as *D_c3J_*, *D_c3K_*, and *D_c3−_*) [28,52], the standard deviation can be decreased to 115.9 kHz, but the *F*_0_ values are unusual for methyl groups (152.4 GHz for one and 164.4 GHz for the other, while typical values range between 158 GHz and 160 GHz) and the *V*_3_ terms are less precisely determined. The molecular parameters obtained with this fit are given in Table S6 in the Supplementary Materials.

The experimental rotational constants were compared with theoretical values. Since the values obtained from *XIAM* and *ntop* differ slightly, we use the experimental values from *XIAM*. This choice is due to the fact that fewer parameters are fitted, resulting in lower correlation and a clearer physical meaning of the rotational constants. All method and basis set combinations mentioned in Section 2.2 produced satisfactory results (see Table S2 in the Supplementary Materials), yielding equilibrium rotational constants in good agreement with the experimental values. An excellent agreement was found between the experimental values and those calculated at the MP2/6-31G(d,p), ωB97X-D/cc-pVDZ, MN15/cc-pVDZ, and MN15/aug-cc-pVDZ levels of theory, with deviations under 0.04%. With these results, we continue to recommend the MP2/6-31G(d,p) level for assignment guidance of small rigid molecules containing a benzene ring. From a theoretical perspective, the experimental rotational constants *B*_0,exp._ should be compared to the zero-point vibrationally corrected rotational constants *B*_0,calc._ rather than to the equilibrium rotational constants *B*_e,calc._. However, performing anharmonic frequency calculations at a sufficiently high level of theory is computationally demanding for an experimental laboratory, and is not necessary, particularly when the primary goal is to obtain predicted values to aid in spectral assignment. For this purpose, we rely on benchmarking studies of equilibrium rotational constants *B*_e,calc._ to identify cost-efficient levels of theory that yield a small *B*_0,exp._-*B*_e,calc._ difference due to error compensation. Our experience suggests that, if a given level of theory provides a small *B*_0,exp._-*B*_e,calc._ difference for one molecule, it often does so for structurally similar molecules, which significantly facilitates initial spectral assignments in new projects.

**Figure 9:**
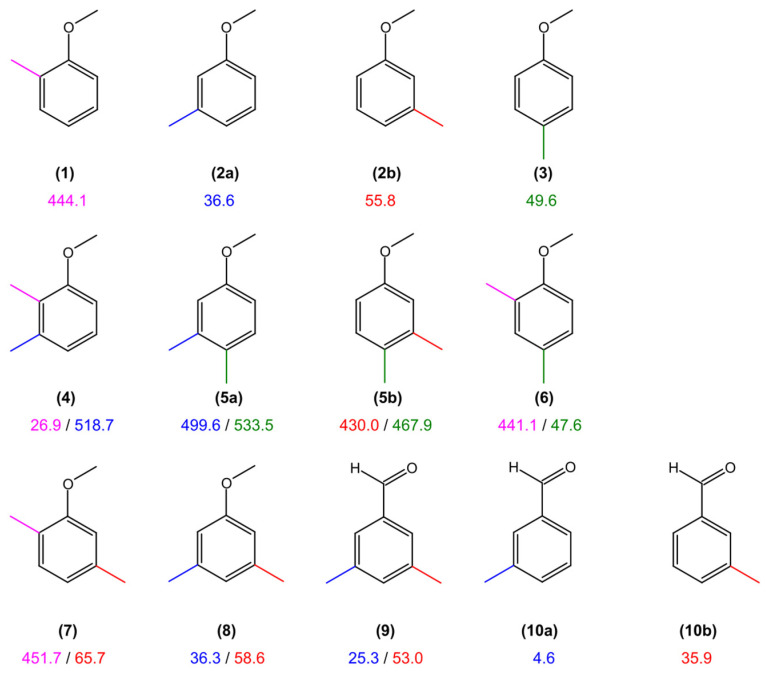
Comparison of the internal rotation barriers of the *syn*-*m* and *anti*-*m*-methyl groups in 35DMA with the values of other toluene derivatives. (**1**) *o*-methylanisole [54], (**2a**) *anti*-*m*-methylanisole [53], (**2b**) *syn*-*m*-methylanisole [53], (**3**) *p*-methylanisole [26], (**4**) 2,3-dimethylanisole [13], (**5a**) *anti*-3,4-dimethylanisole [41], (**5b**) *syn*-3,4-dimethylanisole [41], (**6**) 2,4-dimethylanisole [27], (**7**) 2,5-dimethylanisole [11], (**8**) 3,5-dimethylanisole (this work), (**9**) 3,5-dimethylbenzaldehyde [9], (**10a**) *anti*-*m*-methylbenzaldehyde [55], and (**10b**) *syn*-*m*-methylbenzaldehyde [55].

The torsional barriers of the *syn*-*m* and *anti*-*m*-methyl groups in 35DMA were determined to be 60.1207(56) cm^−1^ and 37.5936(64) cm^−1^, respectively, using the *XIAM* program. Similar values of 58.62367(53) cm^−1^ and 36.28449(69) cm^−1^ were obtained with *ntop*. Compared with the predicted values shown in Table 1, the agreement is reasonably good. When comparing the torsional barriers found for the two *m*-methyl groups in 35DMA (molecule **8** in Figure 9) with other anisole derivatives, we observed that 35DMA is a combination of *anti*-*m* (**2a**) [53] and *syn*-*m*-methylanisole (**2b**) [53]. Specifically, the torsional barrier of 58.6 cm^−1^ for the *syn*-*m*-methyl group in 35DMA (**8**) closely matches the value of *syn*-*m*-methylanisole (55.8 cm^−1^) (**2b**). Similarly, the barrier of 36.3 cm^−1^ for the *anti*-*m*-methyl group is nearly identical to the 36.6 cm^−1^ barrier observed in *anti*-*m*-methylanisole (**2a**). In contrast, a slightly higher value of 65.7 cm^−1^ was obtained for the *m*-methyl group in 2,5-dimethylanisole (**7**) [11]. The difference might arise from coupling between the two rotors, facilitated by electrostatic interactions transmitted through *π*-electron delocalization in the benzene ring. This is supported by the significant *V_cc_* term derived from both the experimental fits (see Table 3) and the 2D-PES calculations (Table S4). Electrostatic effects also explain the slight differences between barrier values for steric-free methyl groups at the *meta*-position and those at the *para*-position in *p*-methylanisole (**3**) (49.6 cm^−1^) [26] and 2,4-dimethylanisole (**6**) (47.6 cm^−1^) [27]. Much higher barriers, ranging from 441.1 cm^−1^ to 451.7 cm^−1^, are observed for methyl groups at the *ortho*-position in *o*-methylanisole (**1**) [54], 2,4-dimethylanisole (**6**) [27], and 2,5-dimethylanisole (**7**) [11] due to steric hindrance. Steric effects also explain the intermediate barriers for the *meta* and *para*-methyl groups in *anti*-3,4-dimethylanisole (**5a**) (499.6 cm^−1^ and 533.5 cm^−1^, respectively) [41] and *syn*-3,4-dimethylanisole (**5b**) (430.0 cm^−1^ and 467.9 cm^−1^, respectively) [41], as well as the *m*-methyl group in 2,3-dimethylanisole (**4**) (518.7 cm^−1^) [13]. An exception to the steric hindrance trend is the remarkably low barrier of 26.9 cm^−1^ for the *o*-methyl group in 2,3-dimethylanisole (**4**), which is squeezed between a methyl group and a methoxy group.

The molecule 3,5-dimethylbenzaldehyde (**9**) [9] is structurally similar to 35DMA (**8**), with two methyl groups in identical positions. Though similar, the barrier heights of the respective methyl groups of 3,5-dimethylbenzaldehyde (**9**) are lower than those of 35DMA (**8**). The discrepancy probably arises from the different electrostatic contributions of the methoxy and aldehyde groups to the π-electron system. This observation is further supported while comparing the barriers for 3,5-dimethylbenzaldehyde (**9**) with those for *anti*-*m*-methylbenzaldehyde (**10a**) [55] and *syn*-*m*-methylbenzaldehyde (**10b**) [55]. Not like in the case of 35DMA, the barriers are very different. Specifically, *anti*-*m*-methylbenzaldehyde (**10a**) has an extremely low barrier of 4.6 cm^−1^, whereas 3,5-dimethylbenzaldehyde (**9**) shows a higher barrier of 25.3 cm^−1^. Similarly, *syn*-*m*-methylbenzaldehyde (**10b**) has a barrier of 35.9 cm^−1^, which is notably lower than the value of 53.0 cm^−1^ for 3,5-dimethylbenzaldehyde (**9**). These differences highlight the significant influence of electronic effects, functional groups, and the coupling between the two rotors on their torsional barriers. Given the limited number of molecules with two methyl groups undergoing internal rotations hindered by low barriers available in the literature, a comprehensive understanding of these effects remains challenging. Additional benchmarking studies on such molecules are needed to confirm and refine the assumptions regarding electrostatic and steric contributions.

## 5. Conclusions

The microwave spectrum of 35DMA was recorded using a pulsed molecular jet Fourier transform microwave spectrometer operating in the frequency range from 2.0 to 26.5 GHz. The high barrier to internal rotation of the methoxy methyl group, exceeding 1000 cm^−1^, resulted in unresolvable torsional splittings in the spectrum. In contrast, the low barrier heights of less than 60 cm^−1^ for the internal rotations of the 3- (*syn*-*m*) and the 5-methyl (*anti*-*m*) groups caused large torsional splittings, with each rotational transition appearing as a quintet. This made the spectral assignment particularly challenging. The initial fits of the five torsional species were performed using the *XIAM* program, but the final fit failed to achieve a standard deviation close to the measurement accuracy of 4 kHz. To verify the assigned frequencies, combination difference loops (Ritz cycles) were employed during the assignment process. Subsequently, separate fits for each torsional species were performed using the *SFLAMS* program, achieving standard deviations of approximately 4 kHz, thereby confirming the correctness of all assigned frequencies. A global fit was then carried out using the *ntop* program, which reduced the deviation from 347.5 kHz (*XIAM*) to 5.3 kHz. The barriers to internal rotation of the *syn-m* and *anti*-*m*-methyl groups were determined to be 58.62367(53) cm^−1^ and 36.28449(69) cm^−1^, respectively. Comparisons with other toluene derivatives containing *meta*-methyl groups suggest that electrostatic effects significantly contribute to the barrier values observed in aromatic molecules.

## Figures and Tables

**Table 1:** Equilibrium rotational constants (*A*_e_, *B*_e_, *C*_e_), vibrational ground state rotational constants (*A*_0_, *B*_0_, *C*_0_), centrifugal distortion constants in the symmetrically reduced Hamiltonian obtained from anharmonic frequency calculations, dipole moment components, and *V*_3_ potential for the *syn*-*m-* and *anti*-*m*-methyl groups at the 3- and 5-positions, respectively, calculated at the MP2/6-31G(d,p), MP2/6-311++G(d,p), and B3LYP-D3BJ/6-311++G(d,p) levels of theory.

Par.	Unit	MP2/6-31G(d,p)	MP2/6-311++G(d,p)	B3LYP-D3BJ
*A*_e_	MHz	1736.4	1728.6	1740.3
*B*_e_	MHz	1095.5	1094.0	1092.6
*C*_e_	MHz	680.3	678.6	679.7
*A*_0_	MHz	1724.2	1717.8	1726.9
*B*_0_	MHz	1085.8	1084.5	1084.0
*C*_0_	MHz	674.9	673.3	674.6
*D_J_*	kHz	0.073437	0.074241	0.074614
*D_JK_*	kHz	−0.104737	−0.105306	−0.097750
*D_K_*	kHz	0.037086	0.036934	0.029037
*d*_1_	kHz	−0.016684	−0.016884	−0.016691
*d*_2_	kHz	0.001187	0.001205	0.002070
|*µ_a_*|	D	0.5	0.5	0.5
|*µ_b_*|	D	1.1	1.2	1.2
|*µ_c_*|	D	0.1	0.0	0.0
*V*_3,*syn-m*_	cm^−1^	55.2	69.2	67.2
*V*_3,*anti-m*_	cm^−1^	36.8	34.6	44.0

**Table 2:** Molecular parameters of the (00), (10), (01), (11), and (12) separate fits obtained with the program *SFLAMS*.

Par. ^a^	Unit	Fit (00)	Fit (10)	Fit (01)	Fit (11)	Fit (12)
*A*	MHz	1744.77418(29)	1744.00056(64)	1735.59250(52)	1734.7419(13)	1735.1469(14)
*B*	MHz	1101.19864(18)	1096.03548(24)	1097.88093(18)	1093.31277(46)	1092.68724(31)
*C*	MHz	680.562851(68)	680.563975(88)	680.531514(74)	680.55101(12)	680.53608(16)
*D_J_*	kHz	0.0286(14)	0.0203(30)	0.0288(24)	0.0278(76)	0.0326(35)
*D_JK_*	kHz	0.1740(66)	0.170(26)	0.330(22)	0.285(60)	0.153(27)
*d*_1_	kHz	−0.01947(87)	−0.0124(14)	−0.0235(11)	−0.0204(39)	−0.0181(19)
*d*_2_	kHz	−0.00821(35)	−0.00525(53)	−0.01183(43)	−0.0092(16)	−0.00443(74)
*q*	MHz		1817.842(14)	1104.1998(14)	1237.8649(25)	881.224(36)
*r*	MHz		563.3230(16)	630.8680(12)	70.043(10)	1127.8147(20)
*q_J_*	kHz		−14.844(99)	−44.003(69)	−55.39(13)	
*q_K_*	kHz		13.52(25)	−21.05(13)	−7.81(17)	
*r_J_*	kHz		−23.138(55)	−15.644(43)	8.18(42)	−34.871(83)
*r_K_*	kHz		−23.90(31)	−120.59(22)	−94.1(22)	−137.82(54)
*N* ^b^		141	148	144	100	89
*rms* ^c^	kHz	3.6	4.8	3.3	4.8	3.0

^a^ All parameters refer to the principal axis system. Waston’s S reduction in I^r^ representation was used. ^b^ Number of lines. ^c^ Root-mean-square deviation of the fit.

**Table 3:** Molecular parameters of 35DMA in the principal axis system obtained using the *XIAM* and *ntop* programs. Rotor 1 refers to the *syn-m* and rotor 2 to the *anti-m*-methyl group.

Par. ^a^	Unit	Fit *XIAM*	Fit *ntop*	Calc. ^b^
*A*	MHz	1737.126(11)	1742.502(62)	1740.3
*B*	MHz	1095.3062(52)	1094.343(15)	1092.6
*C*	MHz	680.5538(17)	680.688(11)	679.7
*D_J_*	kHz		0.01311(38)	0.074614
*D_JK_*	kHz		0.1113(29)	−0.097750
*d*_1_	kHz		−0.00679(22)	−0.016691
*d*_2_	kHz		−0.003364(96)	0.002070
*V_cc_*	cm^−1^	−7.7494(50)	−9.803586(86)	−7.8
*V_ss_*	cm^−1^		0.78428(51)	0.6
*V*_3,1_	cm^−1^	60.1207(56)	58.62367(53)	67.2
*V*_3,2_		37.5936(64)	36.28449(69)	44.0
$D_{\pi^{2}J,1}$	MHz	4.41(71)		
$D_{\pi^{2}K,1}$	MHz	−0.060(11)		
$D_{\pi^{2}-,1}$	MHz	0.0261(22)		
$D_{\pi^{2}J,2}$	MHz		0.02403(75)	
$D_{\pi^{2}K,2}$	MHz	0.3687(78)	−0.5658(79)	
*D_mK_*_,2_	MHz		0.4228(64)	
*d_m_*_,2_	MHz		0.0636(16)	
*V_J_*_,1_	MHz	−44.7(72)	−0.46049(65)	
*V_K_*_,1_	MHz		2.0337(70)	
*V_−_*_,1_	MHz		−0.31988(69)	
*V_J_*_,2_	MHz		1.076(14)	
*V_K_*_,2_	MHz		−10.36(11)	
*V_−_*_,2_	MHz		1.155(15)	
*Q_zJ_*_,1_	kHz		−3.360(26)	
*Q_zK_*_,1_	kHz		8.806(88)	
*Q_z−_*_,1_	kHz		4.014(79)	
*∠*(*i*_1_,*a*)	°	78.0849(22)	78.15036(51)	78.6
*∠*(*i*_1_,*b*)	°	11.9151(22)	11.84964(51)	11.4
*∠*(*i*_1_,*c*)	°	90.0 ^c^	90.0 ^c^	90.0
*∠*(*i*_2_,*a*)	°	41.7799(22)	41.9409(13)	41.5
*∠*(*i*_2_,*b*)	°	131.7799(22)	131.9409(13)	131.5
*∠*(*i*_2_,*c*)	°	90.0 ^c^	90.0 ^c^	90.0
*N* ^d^		622	622	
*rms* ^e^	kHz	347.5	5.3	

^a^ All molecular parameters refer to the principal axis system. Watson’s S reduction in I^r^ representation was used. ^b^ Calculated at the B3LYP-D3BJ/6-311++G(d,p) level of theory. The rotational constants refer to the equilibrium structure. The centrifugal distortion constants are obtained from anharmonic frequency calculations. ^c^ Fixed due to symmetry. ^d^ Number of lines. ^e^ Root-means-square deviation of the fit.

## Data Availability

Data are contained within the article and Supplementary Materials.

## Supplementary Materials

The following Supplementary Materials can be downloaded at: https://www.mdpi.com/article/10.3390/molecules30061195/s1, Table S1: Nuclear coordinates of 35DMA in the principal axis system calculated at the MP2/6-31G(d,p), MP2/6-311++G(d,p), and B3LYP-D3BJ/6-311++G(d,p) levels of theory; Table S2: Rotation constants of 35DMA (in MHz) calculated at different levels of theory; Table S3: Coefficients of Fourier expansion for the one-dimensional potential energy curves of 35DMA given in Figure 3 and Figure 4 calculated at the B3LYP-D3BJ/6-311++G(d,p), MP2/6-311++G(d,p), and MP2/6-31G(d,p) levels of theory; Table S4: Coefficients of the two-dimensional Fourier expansions of the potential energy surfaces of 35DMA given in Figure 5 and Figure 6 calculated at the B3LYP-D3BJ/6-311++G(d,p), MP2/6-311++G(d,p), and MP2/6-31G(d,p) levels of theory; Table S5: Fitted frequencies (ν_obs._) of 35DMA. The ν_obs._ − ν_calc._ residuals are obtained with *XIAM* and *ntop* programs given in Table 3. Table S6: Molecular parameters of 35DMA in the principal axis system obtained using the *XIAM* program by fitting the rotational constants *F*_0,1_ and *F*_0,2_ of the internal rotors.
