# Supplementary material for: Two Coupled Low-Barrier Large Amplitude Motions in 3,5-Dimethylanisole Studied by Microwave Spectroscopy
Source: Molecules. 2025 Mar 7;30(6):1195. doi: 10.3390/molecules30061195 (PMC11945398; doi:10.3390/molecules30061195)
Supplement: Supplementary file 1 [file molecules-30-01195-s001.zip › molecules-3476654-supplementary.pdf]

# Supplementary Materials

## Two coupled low-barrier large amplitude motions in 3,5-dimethylanisole studied by microwave spectroscopy

Safa Khemissi [1]*, Lynn Ferres [2] and Ha Vinh Lam Nguyen [1,3]*

[1] Univ Paris Est Créteil and Université Paris Cité, CNRS, LISA, F-94010 Créteil, France

[2] Institute of Physical Chemistry, RWTH Aachen University, Landoltweg 2, D-52074 Aachen, Germany

[3] Institut Universitaire de France (IUF), F-75231 Paris, France

* Correspondence: safa.khemissi@lisa.ipsl.fr; lam.nguyen@lisa.ipsl.fr

**Table S1.** Nuclear coordinates of 35DMA in the principal axis system calculated at the MP2/6-31G(d,p), MP2/6-311++G(d,p), and B3LYP-D3BJ/6-311++G(d,p) levels of theory. The atoms numbering corresponds to Figure 1.

| | **MP2/6-31G(d,p)** | | | **MP2/6-311++G(d,p)** | | | **B3LYP-D3BJ/6-311++G(d,p)** | | |
|---|---|---|---|---|---|---|---|---|---|
| | ***a*** **/ Å** | ***b*** **/ Å** | ***c*** **/ Å** | ***a*** **/ Å** | ***b*** **/ Å** | ***c*** **/ Å** | ***a*** **/ Å** | ***b*** **/ Å** | ***c*** **/ Å** |
| **C1** | –0.915446 | –0.486833 | –0.000598 | –0.918246 | –0.496326 | 0.012921 | –0.913467 | –0.485392 | 0.000030 |
| **C2** | –0.698707 | 0.891752 | –0.001283 | –0.705784 | 0.886829 | 0.002785 | –0.694267 | 0.889064 | –0.000008 |
| **C3** | 0.613426 | 1.389788 | –0.014634 | 0.606648 | 1.394951 | –0.004178 | 0.618094 | 1.386644 | 0.000002 |
| **C4** | 1.684389 | 0.497461 | –0.015452 | 1.684884 | 0.506536 | –0.022914 | 1.684640 | 0.495925 | 0.000037 |
| **C5** | 1.474929 | –0.889785 | –0.015448 | 1.482062 | –0.885668 | –0.008862 | 1.471927 | –0.891939 | 0.000026 |
| **C6** | 0.169561 | –1.372550 | –0.001449 | 0.176037 | –1.376059 | –0.004487 | 0.170591 | –1.372848 | 0.000028 |
| **C7** | 0.846136 | 2.878227 | 0.013014 | 0.827538 | 2.888977 | 0.010625 | 0.848110 | 2.877680 | –0.000013 |
| **C8** | 2.644541 | –1.838608 | 0.014434 | 2.658852 | –1.831625 | 0.007342 | 2.646229 | –1.838521 | –0.000038 |
| **O9** | –2.152653 | –1.084753 | 0.001225 | –2.149738 | –1.092672 | 0.018691 | –2.149262 | –1.068163 | 0.000003 |
| **C10** | –3.277122 | –0.215332 | 0.003175 | –3.273133 | –0.226924 | –0.015496 | –3.289367 | –0.223523 | –0.000021 |
| **H11** | 0.695968 | 3.282830 | 1.015351 | 0.635453 | 3.305218 | 1.005963 | 0.399528 | 3.346192 | 0.881377 |
| **H12** | 1.862655 | 3.119825 | –0.294377 | 1.857393 | 3.135061 | –0.262791 | 1.913631 | 3.113691 | –0.000284 |
| **H13** | 0.159685 | 3.396302 | –0.656841 | 0.158605 | 3.391574 | –0.695177 | 0.399078 | 3.346225 | –0.881157 |
| **H14** | 3.029569 | –1.956792 | 1.028591 | 3.048340 | –1.954879 | 1.024173 | 3.276396 | –1.682165 | 0.880671 |
| **H15** | 2.359143 | –2.824496 | –0.349981 | 2.374029 | –2.819777 | –0.364725 | 2.318398 | –2.879436 | 0.000415 |
| **H16** | 3.461075 | –1.471546 | –0.606917 | 3.473131 | –1.452378 | –0.617427 | 3.275763 | –1.682771 | –0.881316 |
| **H17** | –4.148007 | –0.863567 | 0.007479 | –4.147978 | –0.876653 | –0.018872 | –4.152697 | –0.886713 | –0.000001 |
| **H18** | –3.289424 | 0.418926 | 0.891936 | –3.304163 | 0.421274 | 0.867897 | –3.317364 | 0.409597 | 0.893594 |
| **H19** | –3.295685 | 0.415234 | –0.888102 | –3.271109 | 0.389777 | –0.921675 | –3.317357 | 0.409559 | –0.893662 |
| **H20** | –1.524968 | 1.589394 | –0.007362 | –1.537089 | 1.584453 | 0.009757 | –1.521624 | 1.586344 | –0.000027 |
| **H21** | 2.698428 | 0.882902 | –0.032926 | 2.700611 | 0.898655 | –0.038007 | 2.700082 | 0.878983 | 0.000070 |
| **H22** | –0.037451 | –2.435703 | –0.007201 | –0.022467 | –2.445090 | –0.005051 | –0.034677 | –2.436729 | 0.000042 |

**Table S2.** Rotation constants of 35DMA (in MHz) calculated at different levels of theory. The difference between the calculated and the experimental (in MHz) are given as Δ*A*, Δ*B,* and Δ*C*. Σ is the sum of the absolute values of Δ*A*, Δ*B,* and Δ*C.* The lower the value of Σ, the closer the three rotational constants are to the experimental constants.

| **Method/Basis set** | ***A*** | **Δ*A*** | ***B*** | **Δ*B*** | ***C*** | **Δ*C*** | **Σ** |
|---|---|---|---|---|---|---|---|
| B3LYP-D3/6-31G(d,p) | 1733.3 | –3.8 | 1088.8 | –6.5 | 677.2 | –3.3 | 13.6 |
| B3LYP-D3/6-31+G(d,p) | 1730.3 | –6.9 | 1085.7 | –9.6 | 675.6 | –5.0 | 21.4 |
| B3LYP-D3/6-31++G(d,p) | 1730.3 | –6.9 | 1085.7 | –9.6 | 675.6 | –5.0 | 21.5 |
| B3LYP-D3/6-311G(d,p) | 1738.0 | 0.9 | 1090.2 | –5.1 | 678.5 | –2.1 | 8.1 |
| B3LYP-D3/6-311+G(d,p) | 1737.3 | 0.2 | 1089.3 | –6.0 | 678.0 | –2.5 | 8.7 |
| B3LYP-D3/6-311++G(d,p) | 1737.4 | 0.3 | 1089.3 | –6.0 | 678.0 | –2.5 | 8.8 |
| B3LYP-D3/6-311G(2d,2p) | 1743.7 | 6.6 | 1094.1 | –1.2 | 680.8 | 0.3 | 8.0 |
| B3LYP-D3/6-311+G(2d,2p) | 1743.2 | 6.0 | 1093.0 | –2.3 | 680.3 | –0.2 | 8.5 |
| B3LYP-D3/6-311++G(2d,2p) | 1743.2 | 6.0 | 1093.0 | –2.3 | 680.3 | –0.2 | 8.6 |
| B3LYP-D3/6-311G(df,pd) | 1744.1 | 6.9 | 1093.7 | –1.6 | 680.7 | 0.2 | 8.7 |
| B3LYP-D3/6-311+G(df,pd) | 1743.6 | 6.4 | 1092.8 | –2.5 | 680.3 | –0.2 | 9.2 |
| B3LYP-D3/6-311++G(df,pd) | 1743.7 | 6.5 | 1092.8 | –2.6 | 680.3 | –0.2 | 9.3 |
| B3LYP-D3/6-311G(2df,2pd) | 1746.5 | 9.3 | 1094.9 | –0.4 | 681.5 | 1.0 | 10.7 |
| B3LYP-D3/6-311+G(2df,2pd) | 1745.8 | 8.7 | 1093.9 | –1.4 | 681.1 | 0.5 | 10.6 |
| B3LYP-D3/6-311++G(2df,2pd) | 1745.8 | 8.7 | 1093.9 | –1.4 | 681.1 | 0.5 | 10.6 |
| B3LYP-D3/6-311G(3df,3pd) | 1746.8 | 9.6 | 1094.9 | –0.4 | 681.6 | 1.0 | 11.1 |
| B3LYP-D3/6-311+G(3df,3pd) | 1746.6 | 9.5 | 1094.3 | –1.1 | 681.3 | 0.8 | 11.3 |
| B3LYP-D3/6-311++G(3df,3pd) | 1746.5 | 9.4 | 1094.3 | –1.1 | 681.3 | 0.8 | 11.2 |
| B3LYP-D3/cc-pVDZ | 1729.2 | –7.9 | 1086.1 | –9.2 | 675.7 | –4.9 | 21.9 |
| B3LYP-D3/cc-pVTZ | 1746.4 | 9.3 | 1094.2 | –1.1 | 681.2 | 0.7 | 11.1 |
| B3LYP-D3/aug-cc-pVDZ | 1729.8 | –7.3 | 1086.0 | –9.3 | 675.7 | –4.8 | 21.4 |
| B3LYP-D3/aug-cc-pVTZ | 1746.3 | 9.2 | 1093.8 | –1.5 | 681.1 | 0.5 | 11.2 |
| | | | | | | | |
| B3LYP-D3BJ/6-31G(d,p) | 1736.3 | –0.8 | 1092.0 | –3.3 | 678.9 | –1.6 | 5.8 |
| B3LYP-D3BJ/6-31+G(d,p) | 1733.2 | –4.0 | 1088.9 | –6.4 | 677.3 | –3.3 | 13.6 |
| B3LYP-D3BJ/6-31++G(d,p) | 1733.2 | –4.0 | 1088.9 | –6.4 | 677.3 | –3.3 | 13.7 |
| B3LYP-D3BJ/6-311G(d,p) | 1740.9 | 3.8 | 1093.5 | –1.9 | 680.2 | –0.4 | 6.0 |
| B3LYP-D3BJ/6-311+G(d,p) | 1740.3 | 3.2 | 1092.5 | –2.8 | 679.7 | –0.8 | 6.8 |
| B3LYP-D3BJ/6-311++G(d,p) | 1740.3 | 3.2 | 1092.6 | –2.7 | 679.7 | –0.8 | 6.7 |
| B3LYP-D3BJ/6-311G(2d,2p) | 1746.6 | 9.5 | 1097.5 | 2.2 | 682.5 | 2.0 | 13.6 |
| B3LYP-D3BJ/6-311+G(2d,2p) | 1746.1 | 9.0 | 1096.3 | 1.0 | 682.0 | 1.5 | 11.5 |
| B3LYP-D3BJ/6-311++G(2d,2p) | 1746.1 | 9.0 | 1096.3 | 1.0 | 682.0 | 1.5 | 11.5 |
| B3LYP-D3BJ/6-311G(df,pd) | 1747.0 | 9.9 | 1096.9 | 1.6 | 682.4 | 1.9 | 13.4 |
| B3LYP-D3BJ/6-311+G(df,pd) | 1746.6 | 9.4 | 1096.0 | 0.7 | 682.0 | 1.5 | 11.6 |
| B3LYP-D3BJ/6-311++G(df,pd) | 1746.6 | 9.4 | 1096.0 | 0.7 | 682.0 | 1.5 | 11.6 |
| B3LYP-D3BJ/6-311G(2df,2pd) | 1749.3 | 12.2 | 1098.2 | 2.9 | 683.2 | 2.7 | 17.8 |
| B3LYP-D3BJ/6-311+G(2df,2pd) | 1748.7 | 11.6 | 1097.2 | 1.9 | 682.8 | 2.2 | 15.6 |

| | | | | | | | |
|---|---|---|---|---|---|---|---|
| B3LYP-D3BJ/6-311++G(2df,2pd) | 1748.7 | 11.6 | 1097.2 | 1.9 | 682.8 | 2.2 | 15.7 |
| B3LYP-D3BJ/6-311G(3df,3pd) | 1749.6 | 12.5 | 1098.2 | 2.9 | 683.3 | 2.7 | 18.1 |
| B3LYP-D3BJ/6-311+G(3df,3pd) | 1749.5 | 12.3 | 1097.6 | 2.2 | 683.0 | 2.5 | 17.0 |
| B3LYP-D3BJ/6-311++G(3df,3pd) | 1749.4 | 12.3 | 1097.5 | 2.2 | 683.0 | 2.5 | 17.0 |
| B3LYP-D3BJ/cc-pVDZ | 1732.2 | –5.0 | 1089.4 | –5.9 | 677.4 | –3.2 | 14.1 |
| B3LYP-D3BJ/cc-pVTZ | 1749.3 | 12.2 | 1097.4 | 2.1 | 683.0 | 2.4 | 16.7 |
| B3LYP-D3BJ/aug-cc-pVDZ | 1732.8 | –4.4 | 1089.3 | –6.0 | 677.4 | –3.1 | 13.5 |
| B3LYP-D3BJ/aug-cc-pVTZ | 1749.2 | 12.1 | 1097.1 | 1.8 | 682.8 | 2.3 | 16.1 |
| | | | | | | | |
| CAM-B3LYP-D3BJ/6-311G(d,p) | 1753.0 | 15.9 | 1101.6 | 6.3 | 685.2 | 4.6 | 26.8 |
| CAM-B3LYP-D3BJ/6-311+G(d,p) | 1752.5 | 15.4 | 1100.7 | 5.4 | 684.7 | 4.2 | 25.0 |
| CAM-B3LYP-D3BJ/6-311++G(d,p) | 1752.5 | 15.3 | 1100.8 | 5.5 | 684.8 | 4.2 | 25.0 |
| CAM-B3LYP-D3BJ/cc-pVDZ | 1743.9 | 6.7 | 1097.4 | 2.1 | 682.3 | 1.7 | 10.6 |
| CAM-B3LYP-D3BJ/cc-pVTZ | 1761.6 | 24.4 | 1105.7 | 10.4 | 688.0 | 7.5 | 42.3 |
| CAM-B3LYP-D3BJ/aug-cc-pVDZ | 1744.7 | 7.6 | 1097.5 | 2.1 | 682.4 | 1.8 | 11.6 |
| CAM-B3LYP-D3BJ/aug-cc-pVTZ | 1761.4 | 24.3 | 1105.4 | 10.1 | 687.9 | 7.3 | 41.7 |
| CCSD/cc-pVDZ | 1715.9 | –21.2 | 1084.6 | –10.7 | 673.1 | –7.4 | 39.4 |
| | | | | | | | |
| M06-2X/6-31G(d,p) | 1744.4 | 7.3 | 1100.0 | 4.7 | 683.3 | 2.7 | 14.7 |
| M06-2X/6-31+G(d,p) | 1741.7 | 4.5 | 1097.6 | 2.3 | 681.9 | 1.4 | 8.2 |
| M06-2X/6-31++G(d,p) | 1741.7 | 4.5 | 1097.6 | 2.3 | 681.9 | 1.4 | 8.2 |
| M06-2X/6-311G(d,p) | 1748.0 | 10.9 | 1101.1 | 5.8 | 684.2 | 3.7 | 20.3 |
| M06-2X/6-311+G(d,p) | 1747.3 | 10.2 | 1100.4 | 5.1 | 683.8 | 3.3 | 18.6 |
| M06-2X/6-311++G(d,p) | 1747.4 | 10.2 | 1100.4 | 5.1 | 683.9 | 3.3 | 18.6 |
| M06-2X/6-311G(2d,2p) | 1753.4 | 16.2 | 1104.7 | 9.4 | 686.4 | 5.8 | 31.4 |
| M06-2X/6-311+G(2d,2p) | 1752.8 | 15.6 | 1103.8 | 8.5 | 686.0 | 5.4 | 29.6 |
| M06-2X/6-311++G(2d,2p) | 1752.8 | 15.7 | 1103.9 | 8.5 | 686.0 | 5.4 | 29.6 |
| M06-2X/6-311G(df,pd) | 1752.9 | 15.8 | 1103.9 | 8.6 | 686.0 | 5.5 | 29.8 |
| M06-2X/6-311+G(df,pd) | 1752.4 | 15.3 | 1103.2 | 7.9 | 685.7 | 5.1 | 28.4 |
| M06-2X/6-311++G(df,pd) | 1752.5 | 15.4 | 1103.2 | 7.9 | 685.7 | 5.2 | 28.5 |
| M06-2X/6-311G(2df,2pd) | 1755.5 | 18.4 | 1105.0 | 9.7 | 686.8 | 6.3 | 34.4 |
| M06-2X/6-311+G(2df,2pd) | 1754.9 | 17.8 | 1104.3 | 9.0 | 686.5 | 5.9 | 32.7 |
| M06-2X/6-311++G(2df,2pd) | 1755.0 | 17.8 | 1104.3 | 9.0 | 686.5 | 5.9 | 32.7 |
| M06-2X/6-311G(3df,3pd) | 1755.5 | 18.3 | 1105.1 | 9.8 | 686.9 | 6.3 | 34.5 |
| M06-2X/6-311+G(3df,3pd) | 1755.3 | 18.1 | 1104.6 | 9.3 | 686.6 | 6.1 | 33.6 |
| M06-2X/6-311++G(3df,3pd) | 1755.3 | 18.1 | 1104.6 | 9.3 | 686.6 | 6.1 | 33.5 |
| M06-2X/cc-pVDZ | 1741.2 | 4.1 | 1098.7 | 3.4 | 682.3 | 1.8 | 9.3 |
| M06-2X/cc-pVTZ | 1755.9 | 18.7 | 1104.7 | 9.4 | 686.8 | 6.2 | 34.3 |
| M06-2X/aug-cc-pVDZ | 1741.9 | 4.8 | 1098.4 | 3.1 | 682.3 | 1.8 | 9.6 |
| M06-2X/aug-cc-pVTZ | 1755.8 | 18.7 | 1104.4 | 9.1 | 686.7 | 6.1 | 34.0 |
| | | | | | | | |
| ωB97X-D/6-31G(d,p) | 1743.8 | 6.7 | 1098.3 | 3.0 | 682.5 | 1.9 | 11.6 |
| ωB97X-D/6-31+G(d,p) | 1741.4 | 4.3 | 1095.8 | 0.5 | 681.2 | 0.7 | 5.5 |
| ωB97X-D/6-31++G(d,p) | 1741.4 | 4.3 | 1095.8 | 0.5 | 681.2 | 0.7 | 5.5 |

| | | | | | | | |
|---|---|---|---|---|---|---|---|
| ωB97X-D/6-311G(d,p) | 1747.9 | 10.8 | 1099.4 | 4.1 | 683.6 | 3.0 | 17.9 |
| ωB97X-D/6-311+G(d,p) | 1747.9 | 10.8 | 1099.4 | 4.1 | 683.6 | 3.0 | 17.9 |
| ωB97X-D/6-311++G(d,p) | 1748.6 | 11.4 | 1100.2 | 4.9 | 684.0 | 3.4 | 19.7 |
| ωB97XD/6-311G(2d,2p) | 1753.7 | 16.5 | 1103.8 | 8.5 | 686.1 | 5.5 | 30.6 |
| ωB97X-D/6-311+G(2d,2p) | 1753.1 | 16.0 | 1102.9 | 7.6 | 685.7 | 5.1 | 28.7 |
| ωB97X-D/6-311++G(2d,2p) | 1753.1 | 16.0 | 1102.9 | 7.6 | 685.7 | 5.1 | 28.7 |
| ωB97X-D/6-311G(df,pd) | 1754.0 | 16.9 | 1103.3 | 8.0 | 686.0 | 5.4 | 30.3 |
| ωB97X-D/6-311+G(df,pd) | 1753.5 | 16.3 | 1102.5 | 7.2 | 685.6 | 5.0 | 28.6 |
| ωB97X-D/6-311++G(df,pd) | 1753.5 | 16.4 | 1102.5 | 7.2 | 685.6 | 5.1 | 28.7 |
| ωB97X-D/6-311G(2df,2pd) | 1756.2 | 19.1 | 1104.4 | 9.1 | 686.7 | 6.1 | 34.2 |
| ωB97X-D/6-311+G(2df,2pd) | 1755.6 | 18.5 | 1103.5 | 8.2 | 686.3 | 5.7 | 32.3 |
| ωB97X-D/6-311++G(2df,2pd) | 1755.6 | 18.5 | 1103.5 | 8.2 | 686.3 | 5.7 | 32.4 |
| ωB97X-D/6-311G(3df,3pd) | 1756.5 | 19.4 | 1104.5 | 9.2 | 686.8 | 6.3 | 34.9 |
| ωB97X-D/6-311+G(3df,3pd) | 1756.3 | 19.1 | 1104.0 | 8.7 | 686.6 | 6.0 | 33.9 |
| ωB97X-D/6-311++G(3df,3pd) | 1756.3 | 19.2 | 1104.0 | 8.7 | 686.6 | 6.0 | 33.9 |
| ωB97X-D/cc-pVDZ | 1739.3 | 2.2 | 1095.7 | 0.4 | 680.9 | 0.4 | 3.0 |
| ωB97X-D/cc-pVTZ | 1756.5 | 19.4 | 1104.1 | 8.8 | 686.6 | 6.1 | 34.3 |
| ωB97X-D/aug-cc-pVDZ | 1740.2 | 3.0 | 1095.8 | 0.5 | 681.1 | 0.5 | 4.1 |
| ωB97X-D/aug-cc-pVTZ | 1756.6 | 19.5 | 1103.9 | 8.6 | 686.6 | 6.0 | 34.1 |
| | | | | | | | |
| MP2/6-31G(d,p) | 1736.4 | –0.7 | 1095.5 | 0.2 | 680.3 | –0.3 | 1.2 |
| MP2/6-31+G(d,p) | 1732.0 | –5.2 | 1091.6 | –3.7 | 678.1 | –2.4 | 11.3 |
| MP2/6-31++G(d,p) | 1731.8 | –5.3 | 1091.4 | –3.9 | 678.0 | –2.5 | 11.7 |
| MP2/6-311G(d,p) | 1730.5 | –6.6 | 1095.6 | 0.3 | 679.5 | –1.1 | 8.0 |
| MP2/6-311+G(d,p) | 1728.7 | –8.4 | 1094.0 | –1.3 | 678.6 | –1.9 | 11.6 |
| MP2/6-311++G(d,p) | 1728.6 | –8.6 | 1094.0 | –1.3 | 678.6 | –2.0 | 11.8 |
| MP2/6-311G(2d,2p) | 1740.7 | 3.6 | 1099.5 | 4.2 | 682.4 | 1.9 | 9.6 |
| MP2/6-311+G(2d,2p) | 1739.1 | 2.0 | 1098.3 | 3.0 | 681.7 | 1.2 | 6.1 |
| MP2/6-311++G(2d,2p) | 1739.2 | 2.1 | 1098.3 | 3.0 | 681.7 | 1.2 | 6.2 |
| MP2/6-311G(df,pd) | 1742.4 | 5.3 | 1104.6 | 9.3 | 684.7 | 4.2 | 18.7 |
| MP2/6-311+G(df,pd) | 1740.5 | 3.4 | 1102.5 | 7.2 | 683.7 | 3.1 | 13.7 |
| MP2/6-311++G(df,pd) | 1740.4 | 3.3 | 1102.6 | 7.3 | 683.7 | 3.1 | 13.7 |
| MP2/6-311G(2df,2pd) | 1749.1 | 12.0 | 1104.9 | 9.6 | 685.8 | 5.2 | 26.8 |
| MP2/6-311+G(2df,2pd) | 1747.7 | 10.6 | 1103.3 | 8.0 | 685.0 | 4.4 | 23.1 |
| MP2/6-311++G(2df,2pd) | 1747.7 | 10.6 | 1103.4 | 8.1 | 685.0 | 4.4 | 23.1 |
| MP2/6-311G(3df,3pd) | 1746.1 | 9.0 | 1102.8 | 7.5 | 684.5 | 4.0 | 20.4 |
| MP2/6-311+G(3df,3pd) | 1745.3 | 8.1 | 1101.9 | 6.6 | 684.1 | 3.5 | 18.3 |
| MP2/6-311++G(3df,3pd) | 1745.4 | 8.3 | 1101.9 | 6.5 | 684.1 | 3.5 | 18.4 |
| MP2-cc-pVDZ | 1717.8 | –19.3 | 1086.9 | –8.4 | 674.3 | –6.3 | 34.0 |
| MP2-cc-pVTZ | 1745.4 | 8.3 | 1102.7 | 7.4 | 684.4 | 3.9 | 19.6 |
| MP2-aug-cc-pVDZ | 1715.1 | –22.0 | 1083.6 | –11.7 | 672.6 | –8.0 | 41.7 |
| MP2-aug-cc-pVTZ | 1744.8 | 7.7 | 1101.3 | 6.0 | 683.8 | 3.2 | 16.9 |
| | | | | | | | |
| MN15/6-31G(d,p) | 1740.8 | 3.7 | 1096.1 | 0.8 | 681.2 | 0.6 | 5.1 |

| | | | | | | | |
|---|---|---|---|---|---|---|---|
| MN15/6-31+G(d,p) | 1737.6 | 0.4 | 1093.2 | –2.1 | 679.6 | –0.9 | 3.4 |
| MN15/6-31++G(d,p) | 1737.5 | 0.4 | 1093.2 | –2.1 | 679.6 | –1.0 | 3.5 |
| MN15/6-311G(d,p) | 1747.3 | 10.1 | 1099.0 | 3.7 | 683.3 | 2.7 | 16.6 |
| MN15/6-311+G(d,p) | 1746.6 | 9.5 | 1098.4 | 3.1 | 682.9 | 2.4 | 15.0 |
| MN15/6-311++G(d,p) | 1746.6 | 9.5 | 1098.4 | 3.1 | 683.0 | 2.4 | 15.0 |
| MN15/6-311G(2d,2p) | 1752.2 | 15.0 | 1103.7 | 8.3 | 685.8 | 5.2 | 28.6 |
| MN15/6-311+G(2d,2p) | 1751.5 | 14.4 | 1102.8 | 7.4 | 685.3 | 4.8 | 26.6 |
| MN15/6-311++G(2d,2p) | 1751.5 | 14.4 | 1102.8 | 7.5 | 685.3 | 4.8 | 26.6 |
| MN15/6-311G(df,pd) | 1754.1 | 17.0 | 1102.9 | 7.6 | 685.8 | 5.3 | 29.9 |
| MN15/6-311+G(df,pd) | 1753.5 | 16.4 | 1102.2 | 6.9 | 685.4 | 4.9 | 28.2 |
| MN15/6-311++G(df,pd) | 1753.5 | 16.3 | 1102.2 | 6.9 | 685.4 | 4.9 | 28.1 |
| MN15/6-311G(2df,2pd) | 1755.1 | 18.0 | 1104.3 | 9.0 | 686.5 | 5.9 | 32.9 |
| MN15/6-311+G(2df,2pd) | 1754.6 | 17.4 | 1103.5 | 8.2 | 686.1 | 5.5 | 31.1 |
| MN15/6-311++G(2df,2pd) | 1754.6 | 17.5 | 1103.5 | 8.2 | 686.1 | 5.5 | 31.2 |
| MN15/6-311G(3df,3pd) | 1756.5 | 19.4 | 1104.6 | 9.3 | 686.8 | 6.3 | 35.0 |
| MN15/6-311+G(3df,3pd) | 1756.2 | 19.0 | 1104.0 | 8.7 | 686.5 | 6.0 | 33.8 |
| MN15/6-311++G(3df,3pd) | 1756.1 | 19.0 | 1104.1 | 8.7 | 686.5 | 6.0 | 33.7 |
| MN15/cc-pVDZ | 1738.2 | 1.0 | 1094.6 | –0.7 | 680.3 | –0.3 | 2.0 |
| MN15/aug-cc-pVDZ | 1739.5 | 2.4 | 1095.6 | 0.3 | 680.9 | 0.3 | 3.0 |
| MN15/cc-pVTZ | 1756.3 | 19.2 | 1104.5 | 9.2 | 686.7 | 6.2 | 34.5 |
| MN15/aug-cc-pVTZ | 1757.0 | 19.9 | 1104.7 | 9.4 | 686.9 | 6.4 | 35.7 |
| | | | | | | | |
| PBE0/6-31G(d,p) | 1719.1 | –18.0 | 1083.4 | –11.9 | 673.1 | –7.5 | 37.3 |
| PBE0/6-31+G(d,p) | 1716.2 | –21.0 | 1080.3 | –15.0 | 671.5 | –9.1 | 45.1 |
| PBE0/6-31++G(d,p) | 1716.2 | –20.9 | 1080.2 | –15.1 | 671.4 | –9.1 | 45.1 |
| PBE0/6-311G(d,p) | 1724.6 | –12.5 | 1085.2 | –10.1 | 674.6 | –5.9 | 28.5 |
| PBE0/6-311+G(d,p) | 1723.9 | –13.2 | 1084.3 | –11.0 | 674.2 | –6.4 | 30.6 |
| PBE0/6-311++G(d,p) | 1724.0 | –13.2 | 1084.3 | –11.0 | 674.2 | –6.4 | 30.5 |
| PBE0/6-311G(2d,2p) | 1729.2 | –8.0 | 1088.9 | –6.4 | 676.7 | –3.9 | 18.2 |
| PBE0/6-311+G(2d,2p) | 1728.7 | –8.4 | 1087.8 | –7.5 | 676.2 | –4.4 | 20.3 |
| PBE0/6-311++G(2d,2p) | 1728.7 | –8.4 | 1087.8 | –7.5 | 676.2 | –4.4 | 20.3 |
| PBE0/6-311G(df,pd) | 1730.2 | –6.9 | 1088.6 | –6.7 | 676.8 | –3.8 | 17.4 |
| PBE0/6-311+G(df,pd) | 1729.6 | –7.5 | 1087.6 | –7.7 | 676.3 | –4.3 | 19.4 |
| PBE0/6-311++G(df,pd) | 1729.6 | –7.5 | 1087.7 | –7.6 | 676.3 | –4.2 | 19.4 |
| PBE0/6-311G(2df,2pd) | 1731.9 | –5.3 | 1089.8 | –5.5 | 677.4 | –3.1 | 13.9 |
| PBE0/6-311+G(2df,2pd) | 1731.3 | –5.8 | 1088.7 | –6.6 | 677.0 | –3.6 | 16.0 |
| PBE0/6-311++G(2df,2pd) | 1731.3 | –5.8 | 1088.7 | –6.6 | 677.0 | –3.6 | 16.0 |
| PBE0/6-311G(3df,3pd) | 1732.4 | –4.7 | 1089.7 | –5.6 | 677.5 | –3.0 | 13.4 |
| PBE0/6-311+G(3df,3pd) | 1732.3 | –4.8 | 1089.1 | –6.2 | 677.3 | –3.3 | 14.3 |
| PBE0/6-311++G(3df,3pd) | 1732.3 | –4.8 | 1089.1 | –6.2 | 677.3 | –3.3 | 14.3 |
| PBE0/cc-pVDZ | 1716.3 | –20.8 | 1081.1 | –14.2 | 671.9 | –8.7 | 43.7 |
| PBE0/aug-cc-pVDZ | 1716.6 | –20.5 | 1081.0 | –14.3 | 671.8 | –8.7 | 43.6 |
| PBE0/cc-pVTZ | 1732.0 | –5.1 | 1089.1 | –6.2 | 677.2 | –3.3 | 14.6 |
| PBE0/aug-cc-pVTZ | 1731.9 | –5.3 | 1088.6 | –6.7 | 677.0 | –3.5 | 15.5 |

| | | | | | | | |
|---|---|---|---|---|---|---|---|
| PBE0-D3/6-31G(d,p) | 1719.6 | –17.5 | 1083.5 | –11.8 | 673.2 | –7.4 | 36.7 |
| PBE0-D3/6-31+G(d,p) | 1716.7 | –20.4 | 1080.4 | –14.9 | 671.6 | –9.0 | 44.3 |
| PBE0-D3/6-31++G(d,p) | 1716.7 | –20.4 | 1080.3 | –15.0 | 671.6 | –9.0 | 44.4 |
| PBE0-D3/6-311G(d,p) | 1725.0 | –12.1 | 1085.4 | –9.9 | 674.8 | –5.8 | 27.8 |
| PBE0-D3/6-311+G(d,p) | 1724.4 | –12.7 | 1084.4 | –10.9 | 674.3 | –6.3 | 29.9 |
| PBE0-D3/6-311++G(d,p) | 1724.5 | –12.7 | 1084.4 | –10.9 | 674.3 | –6.3 | 29.8 |
| PBE0-D3/6-311G(2d,2p) | 1729.6 | –7.6 | 1089.0 | –6.3 | 676.8 | –3.8 | 17.6 |
| PBE0-D3/6-311+G(2d,2p) | 1729.2 | –8.0 | 1087.8 | –7.5 | 676.3 | –4.3 | 19.7 |
| PBE0-D3/6-311++G(2d,2p) | 1729.2 | –8.0 | 1087.8 | –7.5 | 676.3 | –4.3 | 19.7 |
| PBE0-D3/6-311G(df,pd) | 1730.6 | –6.5 | 1088.7 | –6.6 | 676.9 | –3.7 | 16.7 |
| PBE0-D3/6-311+G(df,pd) | 1730.0 | –7.1 | 1087.7 | –7.6 | 676.4 | –4.1 | 18.8 |
| PBE0-D3/6-311++G(df,pd) | 1730.1 | –7.0 | 1087.8 | –7.6 | 676.4 | –4.1 | 18.7 |
| PBE0-D3/6-311G(2df,2pd) | 1732.3 | –4.8 | 1089.8 | –5.5 | 677.5 | –3.0 | 13.3 |
| PBE0-D3/6-311+G(2df,2pd) | 1731.8 | –5.4 | 1088.8 | –6.5 | 677.1 | –3.5 | 15.4 |
| PBE0-D3/6-311++G(2df,2pd) | 1731.8 | –5.3 | 1088.8 | –6.5 | 677.1 | –3.5 | 15.4 |
| PBE0-D3/6-311G(3df,3pd) | 1732.9 | –4.3 | 1089.8 | –5.5 | 677.6 | –2.9 | 12.7 |
| PBE0-D3/6-311+G(3df,3pd) | 1732.8 | –4.4 | 1089.2 | –6.1 | 677.4 | –3.2 | 13.7 |
| PBE0-D3/6-311++G(3df,3pd) | 1732.8 | –4.3 | 1089.2 | –6.1 | 677.4 | –3.2 | 13.7 |
| PBE0-D3/cc-pVDZ | 1716.8 | –20.3 | 1081.2 | –14.1 | 672.0 | –8.6 | 43.0 |
| PBE0-D3/aug-cc-pVDZ | 1717.1 | –20.0 | 1081.0 | –14.3 | 671.9 | –8.6 | 42.9 |
| PBE0-D3/cc-pVTZ | 1732.5 | –4.6 | 1089.2 | –6.1 | 677.3 | –3.2 | 14.0 |
| PBE0-D3/aug-cc-pVTZ | 1732.3 | –4.8 | 1088.7 | –6.6 | 677.1 | –3.4 | 14.8 |
| **Experiemental** | **1737.1** | | **1095.3** | | **680.6** | | |

**Table S3.** Coefficients of Fourier expansion for the one-dimensional potential energy curves of the 35DMA given in Figures 3 and 4 calculated at the B3LYP-D3BJ/6-311++G(d,p), MP2/6-311++G(d,p) and MP2/6-31G(d,p) levels of theory. The potential is expanded as $V(\alpha) = \sum_{i=0}^{n} a_i f_i$.

| **B3LYP-D3BJ/6-311++G(d,p)** | | | | |
|---|---|---|---|---|
| | ***anti-m*** | | ***syn-m*** | |
| $f_i$ | $a_i$ / Hartree | $a_i$ / cm$^{-1}$ | $a_i$ / Hartree | $a_i$ / cm$^{-1}$ |
| 1 | –425.5591774 | | –425.5591291 | |
| cos(3$\alpha$) | 0.000100402 | 22.0 | –0.000153178 | –33.6 |
| cos(6$\alpha$) | 0.000011161 | 2.4 | 0.000016422 | 3.6 |

| **MP2/6-311++G(d,p)** | | | | |
|---|---|---|---|---|
| | ***anti-m*** | | ***syn-m*** | |
| $f_i$ | $a_i$ / Hartree | $a_i$ / cm$^{-1}$ | $a_i$ / Hartree | $a_i$ / cm$^{-1}$ |
| 1 | –424.2440596 | | –424.2440212 | |
| cos(3$\alpha$) | 0.000078616 | 17.3 | –0.000157538 | –34.6 |
| cos(6$\alpha$) | 0.000059677 | 13.1 | 0.000066852 | 14.7 |
| cos(9$\alpha$) | – | – | –0.000004866 | –1.1 |
| cos(12$\alpha$) | 0.000002328 | 0.5 | 0.000002432 | 0.5 |
| sin(3$\alpha$) | –0.000010269 | –2.3 | 0.000021471 | 4.7 |
| sin(6$\alpha$) | 0.000003434 | 0.8 | –0.000005136 | –1.1 |

| **MP2/6-31G(d,p)** | | | | |
|---|---|---|---|---|
| | ***anti-m*** | | ***syn-m*** | |
| $f_i$ | $a_i$ / Hartree | $a_i$ / cm$^{-1}$ | $a_i$ / Hartree | $a_i$ / cm$^{-1}$ |
| 1 | –424.0833469 | | –424.0833272 | |
| cos(3$\alpha$) | 0.000083747 | 18.4 | –0.000125847 | –27.6 |
| cos(6$\alpha$) | 0.000049899 | 11.0 | 0.000059847 | 13.1 |
| cos(9$\alpha$) | –0.000002456 | –0.5 | –0.000003914 | –0.9 |
| sin(3$\alpha$) | –0.000002154 | –0.5 | 0.000003895 | 0.9 |
| sin(6$\alpha$) | – | – | –0.000001165 | –0.3 |

**Table S4.** Coefficients of the two-dimensional Fourier expansions of the potential energy surfaces of 35DMA given in Figures 5 and 6 obtained by varying the dihedral angles $\alpha_1 = \angle(C_2, C_3, C_7, H_{12})$ and $\alpha_2 = \angle(C_4, C_5, C_8, H_{15})$ in a grid of 10°. The calculations were carried out at the B3LYP-D3BJ/6-311++G(d,p), MP2/6-311++G(d,p) and MP2/6-31G(d,p) levels of theory. The potentials are expanded as $V(\alpha) = \sum_{i=0}^{n} a_i f_i$.

| | | **B3LYP-D3BJ/ 6-311++G(d,p)** | | **MP2/ 6-311++G(d,p)** | | **MP2/ 6-31G(d,p)** | |
|---|---|---|---|---|---|---|---|
| i | $f_i$ | $a_i$ / Hartree | $a_i$ / $cm^{-1}$ | $a_i$ / Hartree | $a_i$ / $cm^{-1}$ | $a_i$ / Hartree | $a_i$ / $cm^{-1}$ |
| 0 | 1 | –425.5590741 | | –424.2439529 | | –424.0832616 | |
| 1 | $\cos(3\alpha_1)$ | –0.000118534 | –26.0 | –0.000149001 | –32.7 | –0.000115954 | –25.4 |
| 2 | $\cos(3\alpha_2)$ | 0.000065427 | 14.4 | 0.000060756 | 13.3 | 0.000068252 | 15.0 |
| 3 | $\cos(6\alpha_1)$ | 0.000016133 | 3.5 | 0.000068828 | 15.1 | 0.000060153 | 13.2 |
| 4 | $\cos(6\alpha_2)$ | 0.000011633 | 2.6 | 0.000060403 | 13.3 | 0.000050997 | 11.2 |
| 5 | $\cos(3\alpha_1)\cos(3\alpha_2)$ | –0.000035437 | –7.8 | –0.000030256 | –6.6 | –0.000030523 | –6.7 |
| 6 | $\sin(3\alpha_1)\sin(3\alpha_2)$ | 0.000002727 | 0.6 | 0.000017853 | 3.9 | 0.000002884 | 0.6 |
| 7 | $\cos(6\alpha_1)\cos(3\alpha_2)$ | – | – | –0.000001562 | –0.3 | – | – |
| 8 | $\sin(6\alpha_1)\sin(3\alpha_2)$ | – | – | 0.000004296 | 0.9 | – | – |
| 9 | $\sin(3\alpha_1)\sin(6\alpha_2)$ | – | – | 0.000006269 | 1.4 | – | – |
| 10 | $\cos(6\alpha_1)\cos(6\alpha_2)$ | – | – | 0.000002163 | 0.5 | – | – |

**Table S5.** Fitted frequencies ($\nu_{obs}$) of 35DMA. The $\nu_{obs} - \nu_{calc}$ residuals are obtained with *XIAM* and *ntop* programs.

| $J'$ | $K_a'$ | $K_c'$ | $J$ | $K_a$ | $K_c$ | Species | $\nu_{obs}$ | $\nu_{obs} - \nu_{calc}$ | $\nu_{obs} - \nu_{calc}$ |
|---|---|---|---|---|---|---|---|---|---|
| Upper level | | | Lower level | | | | MHz | *XIAM* / kHz | *ntop* / kHz |
| 2 | 0 | 2 | 1 | 0 | 1 | (00) | 3414.6090 | 66.6 | 1.2 |
| 2 | 0 | 2 | 1 | 0 | 1 | (10) | 3267.7050 | –142.2 | –3.9 |
| 2 | 0 | 2 | 1 | 0 | 1 | (01) | 3213.3344 | –200.2 | 1.3 |
| 2 | 0 | 2 | 1 | 0 | 1 | (11) | 3415.2947 | –151.3 | 0.7 |
| 2 | 1 | 1 | 1 | 1 | 0 | (00) | 3984.1579 | 130.6 | 1.4 |
| 2 | 1 | 1 | 1 | 1 | 0 | (10) | 3825.0208 | –41.4 | –2.9 |
| 2 | 1 | 2 | 1 | 0 | 1 | (10) | 3923.6445 | 99.0 | 1.3 |
| 2 | 1 | 2 | 1 | 0 | 1 | (11) | 3701.6449 | 425.7 | 1.1 |
| 2 | 2 | 1 | 1 | 1 | 0 | (00) | 5914.8765 | –70.2 | –3.7 |
| 2 | 2 | 1 | 1 | 1 | 0 | (10) | 5515.3396 | 353.3 | –6.6 |
| 2 | 2 | 1 | 1 | 1 | 0 | (01) | 3921.9151 | 499.8 | 9.9 |
| 3 | 0 | 3 | 2 | 0 | 2 | (00) | 4841.7808 | 56.3 | –0.9 |
| 3 | 0 | 3 | 2 | 0 | 2 | (10) | 4860.2735 | –32.1 | –1.3 |
| 3 | 0 | 3 | 2 | 0 | 2 | (01) | 4861.4970 | –52.1 | 1.4 |
| 3 | 0 | 3 | 2 | 0 | 2 | (11) | 4838.5358 | 136.2 | 0.1 |
| 3 | 0 | 3 | 2 | 0 | 2 | (12) | 4705.3176 | 207.5 | –3.4 |
| 3 | 0 | 3 | 2 | 1 | 2 | (00) | 4469.9276 | 124.8 | –0.7 |
| 3 | 0 | 3 | 2 | 1 | 2 | (10) | 4204.3349 | –272.4 | –5.5 |
| 3 | 0 | 3 | 2 | 1 | 2 | (01) | 4160.8064 | –307.2 | 2.8 |
| 3 | 0 | 3 | 2 | 1 | 2 | (11) | 4552.1864 | –440.0 | 0.4 |
| 3 | 1 | 2 | 2 | 1 | 1 | (00) | 5859.5403 | 164.6 | 0.1 |
| 3 | 1 | 2 | 2 | 1 | 1 | (10) | 5422.4790 | –323.1 | –3.1 |
| 3 | 1 | 2 | 2 | 1 | 1 | (01) | 5255.5369 | –558.6 | –1.8 |
| 3 | 1 | 2 | 2 | 1 | 1 | (11) | 5772.5917 | –404.6 | –1.0 |
| 3 | 1 | 2 | 2 | 1 | 1 | (12) | 4761.6325 | 351.9 | 8.5 |
| 3 | 1 | 2 | 2 | 2 | 1 | (10) | 3732.1575 | –720.5 | –2.1 |
| 3 | 1 | 2 | 2 | 2 | 1 | (11) | 4671.2724 | –732.9 | –4.3 |
| 3 | 1 | 3 | 2 | 0 | 2 | (00) | 5005.0111 | 7.9 | –0.9 |
| 3 | 1 | 3 | 2 | 0 | 2 | (10) | 5216.1203 | 156.2 | 3.7 |
| 3 | 1 | 3 | 2 | 0 | 2 | (01) | 5260.6975 | 137.3 | 0.3 |
| 3 | 1 | 3 | 2 | 0 | 2 | (11) | 4969.9164 | 420.8 | –0.1 |
| 3 | 1 | 3 | 2 | 0 | 2 | (12) | 5577.1446 | –196.3 | 6.4 |
| 3 | 1 | 3 | 2 | 1 | 2 | (00) | 4633.1578 | 76.3 | –0.7 |
| 3 | 1 | 3 | 2 | 1 | 2 | (10) | 4560.1808 | –85.0 | –1.5 |
| 3 | 1 | 3 | 2 | 1 | 2 | (01) | 4560.0051 | –119.6 | 0.0 |
| 3 | 1 | 3 | 2 | 1 | 2 | (11) | 4683.5670 | –155.4 | 0.2 |
| 3 | 2 | 1 | 2 | 1 | 2 | (00) | 9190.3228 | 165.0 | 3.0 |
| 3 | 2 | 1 | 2 | 1 | 2 | (10) | 9804.3231 | 139.7 | 8.3 |
| 3 | 2 | 1 | 2 | 1 | 2 | (01) | 10341.6645 | 906.5 | 7.0 |

| | | | | | | | | | |
|---|---|---|---|---|---|---|---|---|---|
| 3 | 2 | 1 | 2 | 1 | 2 | (11) | 9673.6269 | –0.4 | 0.8 |
| 3 | 2 | 1 | 2 | 2 | 0 | (00) | 5848.7835 | 233.4 | 7.1 |
| 3 | 2 | 1 | 2 | 2 | 0 | (10) | 5883.1625 | 515.6 | 3.4 |
| 3 | 2 | 2 | 2 | 1 | 1 | (00) | 7276.0155 | –41.0 | 13.6 |
| 3 | 2 | 2 | 2 | 1 | 1 | (10) | 7098.8007 | 71.9 | –1.5 |
| 3 | 2 | 2 | 2 | 1 | 1 | (01) | 6717.6049 | –362.2 | 0.4 |
| 3 | 2 | 2 | 2 | 1 | 1 | (11) | 6670.8952 | 547.5 | 0.4 |
| 3 | 2 | 2 | 2 | 2 | 1 | (00) | 5345.2864 | 149.2 | 8.2 |
| 3 | 2 | 2 | 2 | 2 | 1 | (10) | 5408.4804 | –324.4 | 0.6 |
| 3 | 2 | 2 | 2 | 2 | 1 | (01) | 5680.5623 | 37.7 | 0.3 |
| 3 | 2 | 2 | 2 | 2 | 1 | (11) | 5569.5751 | 218.4 | –3.7 |
| 3 | 2 | 2 | 2 | 2 | 1 | (12) | 5449.7739 | 240.0 | 14.9 |
| **3** | **3** | **0** | **2** | **2** | **0** | **(10)** | **9775.2242** | **–642.2** | **–7.1** |
| **3** | **3** | **0** | **2** | **2** | **0** | **(01)** | **10702.2239** | **–1257.7** | **1.3** |
| **3** | **3** | **0** | **2** | **2** | **0** | **(11)** | **10822.9417** | **0.4** | **4.9** |
| 3 | 3 | 0 | 2 | 2 | 1 | (00) | 9731.4248 | –77.0 | 0.0 |
| 3 | 3 | 0 | 2 | 2 | 1 | (10) | 10509.5359 | –1501.5 | –1.6 |
| 3 | 3 | 1 | 2 | 2 | 0 | (00) | 9546.9892 | –120.0 | –0.1 |
| 3 | 3 | 1 | 2 | 2 | 0 | (10) | 8715.6567 | 877.5 | –11.1 |
| 3 | 3 | 1 | 2 | 2 | 0 | (01) | 5642.0108 | 282.6 | 9.3 |
| 4 | 0 | 4 | 3 | 0 | 3 | (00) | 6169.2368 | 60.5 | –0.4 |
| 4 | 0 | 4 | 3 | 0 | 3 | (10) | 6234.8030 | 40.3 | –0.3 |
| 4 | 0 | 4 | 3 | 0 | 3 | (01) | 6255.7601 | 38.3 | 0.0 |
| 4 | 0 | 4 | 3 | 0 | 3 | (11) | 6162.7366 | 128.0 | 0.2 |
| 4 | 0 | 4 | 3 | 0 | 3 | (12) | 6303.3785 | 40.3 | 1.0 |
| 4 | 0 | 4 | 3 | 1 | 3 | (00) | 6006.0068 | 109.2 | –0.2 |
| 4 | 0 | 4 | 3 | 1 | 3 | (10) | 5878.9576 | –146.7 | –3.8 |
| 4 | 0 | 4 | 3 | 1 | 3 | (01) | 5856.5604 | –150.3 | 1.9 |
| 4 | 0 | 4 | 3 | 1 | 3 | (11) | 6031.3562 | –156.4 | 0.5 |
| 4 | 0 | 4 | 3 | 1 | 3 | (12) | 5431.5528 | 445.4 | –7.5 |
| 4 | 1 | 3 | 3 | 1 | 2 | (00) | 7555.0010 | 156.0 | 1.0 |
| 4 | 1 | 3 | 3 | 1 | 2 | (10) | 7223.4380 | –347.6 | –1.6 |
| 4 | 1 | 3 | 3 | 1 | 2 | (01) | 7130.5113 | –383.9 | –0.3 |
| 4 | 1 | 3 | 3 | 1 | 2 | (11) | 7502.8630 | –248.2 | –1.0 |
| 4 | 1 | 3 | 3 | 1 | 2 | (12) | 6583.6643 | 478.1 | 0.7 |
| **4** | **1** | **3** | **3** | **1** | **3** | **(10)** | **9582.2667** | **–496.5** | **0.2** |
| 4 | 1 | 3 | 3 | 2 | 2 | (00) | 6138.5376 | 373.3 | –0.8 |
| 4 | 1 | 3 | 3 | 2 | 2 | (10) | 5547.1178 | –741.0 | –1.6 |
| 4 | 1 | 3 | 3 | 2 | 2 | (01) | 5668.4449 | –578.7 | –0.9 |
| 4 | 1 | 3 | 3 | 2 | 2 | (11) | 6604.5606 | –1199.1 | –1.2 |
| 4 | 1 | 4 | 3 | 0 | 3 | (00) | 6228.4486 | 35.3 | –0.8 |
| 4 | 1 | 4 | 3 | 0 | 3 | (10) | 6380.5334 | 140.2 | 2.3 |
| 4 | 1 | 4 | 3 | 0 | 3 | (01) | 6421.8225 | 124.8 | –1.7 |
| 4 | 1 | 4 | 3 | 0 | 3 | (11) | 6210.7133 | 230.5 | –0.2 |

| | | | | | | | | | |
|---|---|---|---|---|---|---|---|---|---|
| 4 | 1 | 4 | 3 | 0 | 3 | (12) | 6767.5977 | –288.5 | 7.7 |
| 4 | 1 | 4 | 3 | 1 | 3 | (00) | 6065.2187 | 84.1 | –0.5 |
| 4 | 1 | 4 | 3 | 1 | 3 | (10) | 6024.6874 | –47.3 | –1.9 |
| 4 | 1 | 4 | 3 | 1 | 3 | (01) | 6022.6221 | –64.5 | –0.5 |
| 4 | 1 | 4 | 3 | 1 | 3 | (11) | 6079.3326 | –54.2 | –0.1 |
| 4 | 1 | 4 | 3 | 1 | 3 | (12) | 5895.7715 | 116.1 | –1.3 |
| **4** | **2** | **2** | **3** | **1** | **2** | **(01)** | **10922.2826** | **443.0** | **5.3** |
| **4** | **2** | **2** | **3** | **1** | **2** | **(11)** | **10176.5950** | **674.9** | **–0.9** |
| 4 | 2 | 2 | 3 | 1 | 3 | (00) | 12515.5615 | 373.0 | 3.1 |
| 4 | 2 | 2 | 3 | 1 | 3 | (10) | 12995.9522 | 234.7 | 2.8 |
| 4 | 2 | 2 | 3 | 1 | 3 | (01) | 13273.1317 | 320.8 | 10.4 |
| 4 | 2 | 2 | 3 | 1 | 3 | (11) | 12657.6248 | 380.2 | –2.7 |
| 4 | 2 | 2 | 3 | 2 | 1 | (00) | 7958.3962 | 284.0 | –0.9 |
| 4 | 2 | 2 | 3 | 2 | 1 | (10) | 7751.8189 | 19.1 | 2.1 |
| 4 | 2 | 2 | 3 | 2 | 1 | (01) | 7491.4642 | –713.5 | –4.7 |
| 4 | 2 | 2 | 3 | 2 | 1 | (11) | 7667.5666 | 226.9 | –1.5 |
| **4** | **2** | **2** | **3** | **2** | **2** | **(01)** | **9460.2140** | **246.0** | **2.5** |
| 4 | 2 | 3 | 3 | 1 | 2 | (00) | 8424.0165 | –59.9 | –2.0 |
| 4 | 2 | 3 | 3 | 1 | 2 | (10) | 8599.9487 | 145.6 | 3.5 |
| 4 | 2 | 3 | 3 | 1 | 2 | (01) | 8515.2775 | –33.8 | 1.7 |
| 4 | 2 | 3 | 3 | 1 | 2 | (11) | 8132.4173 | 809.7 | –0.7 |
| 4 | 2 | 3 | 3 | 1 | 2 | (12) | 8777.0550 | 51.8 | 2.9 |
| 4 | 2 | 3 | 3 | 2 | 2 | (00) | 7007.5564 | 160.8 | –0.4 |
| 4 | 2 | 3 | 3 | 2 | 2 | (10) | 6923.6271 | –249.2 | 2.0 |
| 4 | 2 | 3 | 3 | 2 | 2 | (01) | 7053.2071 | –232.5 | –3.0 |
| 4 | 2 | 3 | 3 | 2 | 2 | (11) | 7234.1132 | –142.9 | –2.6 |
| **4** | **3** | **1** | **3** | **2** | **1** | **(10)** | **11431.3446** | **–719.2** | **–9.5** |
| **4** | **3** | **1** | **3** | **2** | **1** | **(01)** | **11902.2887** | **100.1** | **–1.1** |
| **4** | **3** | **1** | **3** | **2** | **1** | **(11)** | **11785.3760** | **–128.1** | **1.3** |
| 4 | 3 | 1 | 3 | 2 | 2 | (00) | 11926.4778 | 62.9 | –0.2 |
| 4 | 3 | 1 | 3 | 2 | 2 | (10) | 12640.3354 | –741.7 | –4.3 |
| 4 | 3 | 1 | 3 | 3 | 0 | (00) | 7540.3386 | 288.4 | 7.2 |
| **4** | **3** | **2** | **3** | **1** | **2** | **(10)** | **13230.1384** | **801.5** | **0.9** |
| **4** | **3** | **2** | **3** | **1** | **2** | **(01)** | **12502.6737** | **–379.3** | **12.8** |
| 4 | 3 | 2 | 3 | 2 | 1 | (00) | 11045.7882 | –129.3 | –3.2 |
| 4 | 3 | 2 | 3 | 2 | 1 | (10) | 10344.8301 | 433.2 | –1.7 |
| 4 | 3 | 2 | 3 | 2 | 1 | (01) | 9071.8612 | –1529.9 | 8.6 |
| **4** | **3** | **2** | **3** | **2** | **2** | **(10)** | **11553.8202** | **410.0** | **2.9** |
| 4 | 3 | 2 | 3 | 3 | 1 | (00) | 7347.5843 | 225.8 | 5.8 |
| 4 | 3 | 2 | 3 | 3 | 1 | (10) | 7512.3383 | 73.7 | 15.3 |
| 4 | 3 | 2 | 3 | 3 | 1 | (01) | 7752.7621 | 609.0 | 9.2 |
| 4 | 3 | 2 | 3 | 3 | 1 | (11) | 7441.4325 | 257.7 | –6.8 |
| **4** | **4** | **0** | **3** | **3** | **0** | **(10)** | **13292.2601** | **–667.0** | **–12.8** |
| 4 | 4 | 0 | 3 | 3 | 1 | (00) | 13149.5248 | –152.3 | 3.3 |

| | | | | | | | | | |
|---|---|---|---|---|---|---|---|---|---|
| 4 | 4 | 1 | 3 | 3 | 0 | (00) | 13107.0452 | –168.1 | 3.2 |
| **4** | **4** | **1** | **3** | **3** | **1** | **(01)** | **12478.4552** | **–1408.3** | **0.6** |
| **4** | **4** | **1** | **3** | **3** | **1** | **(11)** | **12643.1632** | **–7.9** | **0.5** |
| 5 | 0 | 5 | 4 | 0 | 4 | (00) | 7498.2064 | 76.0 | –0.6 |
| 5 | 0 | 5 | 4 | 0 | 4 | (10) | 7536.8523 | 35.9 | –1.2 |
| 5 | 0 | 5 | 4 | 0 | 4 | (01) | 7551.0208 | 28.9 | –1.1 |
| 5 | 0 | 5 | 4 | 0 | 4 | (11) | 7491.8493 | 66.4 | 0.3 |
| 5 | 0 | 5 | 4 | 0 | 4 | (12) | 7656.8029 | –101.9 | 2.7 |
| 5 | 0 | 5 | 4 | 1 | 4 | (00) | 7438.9942 | 100.8 | –0.6 |
| 5 | 0 | 5 | 4 | 1 | 4 | (10) | 7391.1217 | –64.2 | –3.9 |
| 5 | 0 | 5 | 4 | 1 | 4 | (01) | 7384.9590 | –57.0 | 1.2 |
| 5 | 0 | 5 | 4 | 1 | 4 | (11) | 7443.8723 | –36.3 | 0.4 |
| 5 | 0 | 5 | 4 | 1 | 4 | (12) | 7192.5837 | 226.9 | –3.9 |
| 5 | 1 | 4 | 4 | 1 | 3 | (00) | 9017.0616 | 111.3 | –0.2 |
| 5 | 1 | 4 | 4 | 1 | 3 | (10) | 8973.3412 | –188.7 | –0.2 |
| 5 | 1 | 4 | 4 | 1 | 3 | (01) | 8951.4500 | –176.5 | 4.0 |
| 5 | 1 | 4 | 4 | 1 | 3 | (11) | 8980.4686 | 201.2 | –0.6 |
| 5 | 1 | 4 | 4 | 1 | 3 | (12) | 8565.5377 | 497.2 | 0.4 |
| 5 | 1 | 4 | 4 | 2 | 3 | (00) | 8148.0432 | 324.2 | –0.1 |
| 5 | 1 | 4 | 4 | 2 | 3 | (10) | 7596.8309 | –681.5 | –4.9 |
| 5 | 1 | 4 | 4 | 2 | 3 | (01) | 7566.6875 | –523.0 | 5.8 |
| 5 | 1 | 4 | 4 | 2 | 3 | (11) | 8350.9155 | –855.5 | 0.3 |
| 5 | 1 | 4 | 4 | 2 | 3 | (12) | 6372.1468 | 923.3 | –2.0 |
| 5 | 1 | 5 | 4 | 0 | 4 | (00) | 7517.4084 | 65.4 | –0.8 |
| 5 | 1 | 5 | 4 | 0 | 4 | (10) | 7587.2016 | 77.6 | 0.2 |
| 5 | 1 | 5 | 4 | 0 | 4 | (01) | 7608.2152 | 57.2 | –1.9 |
| 5 | 1 | 5 | 4 | 0 | 4 | (11) | 7507.4361 | 97.7 | –0.2 |
| 5 | 1 | 5 | 4 | 0 | 4 | (12) | 7846.5709 | –276.4 | 5.6 |
| 5 | 1 | 5 | 4 | 1 | 4 | (00) | 7458.1966 | 90.6 | –0.3 |
| 5 | 1 | 5 | 4 | 1 | 4 | (10) | 7441.4713 | –22.2 | –2.3 |
| 5 | 1 | 5 | 4 | 1 | 4 | (01) | 7442.1528 | –29.3 | –0.2 |
| 5 | 1 | 5 | 4 | 1 | 4 | (11) | 7459.4595 | –4.7 | 0.2 |
| 5 | 1 | 5 | 4 | 1 | 4 | (12) | 7382.3521 | 52.7 | –0.6 |
| **5** | **2** | **3** | **4** | **1** | **3** | **(11)** | **12420.3041** | **575.5** | **–6.3** |
| 5 | 2 | 3 | 4 | 1 | 4 | (10) | 16338.7240 | –181.6 | 12.3 |
| 5 | 2 | 3 | 4 | 1 | 4 | (11) | 16324.8611 | 83.6 | –12.2 |
| 5 | 2 | 3 | 4 | 1 | 4 | (12) | 16576.2877 | 101.8 | –13.1 |
| 5 | 2 | 3 | 4 | 2 | 2 | (00) | 9939.8246 | 294.3 | –1.0 |
| 5 | 2 | 3 | 4 | 2 | 2 | (10) | 9367.4545 | –468.4 | 2.9 |
| 5 | 2 | 3 | 4 | 2 | 2 | (01) | 9127.8661 | –734.8 | –2.7 |
| 5 | 2 | 3 | 4 | 2 | 2 | (11) | 9746.5724 | –347.3 | –6.1 |
| 5 | 2 | 3 | 4 | 2 | 2 | (12) | 8576.9789 | 521.9 | 25.8 |
| **5** | **2** | **3** | **4** | **2** | **3** | **(10)** | **11404.6358** | **–223.9** | **6.9** |
| **5** | **2** | **3** | **4** | **2** | **3** | **(01)** | **11534.8715** | **–257.8** | **1.3** |

| | | | | | | | | | |
|---|---|---|---|---|---|---|---|---|---|
| **5** | **2** | **3** | **4** | **2** | **3** | **(12)** | **11322.2646** | **223.7** | **–8.3** |
| 5 | 2 | 3 | 4 | 3 | 2 | (00) | 6852.4168 | 691.9 | –14.5 |
| 5 | 2 | 3 | 4 | 3 | 2 | (11) | 7979.3639 | –689.0 | –3.5 |
| 5 | 2 | 3 | 4 | 3 | 2 | (12) | 6556.0684 | 156.4 | –3.2 |
| 5 | 2 | 4 | 4 | 1 | 3 | (00) | 9450.5504 | –43.7 | 4.1 |
| 5 | 2 | 4 | 4 | 1 | 3 | (10) | 9850.5569 | 318.7 | 5.7 |
| 5 | 2 | 4 | 4 | 1 | 3 | (01) | 9889.5554 | 140.3 | 2.5 |
| 5 | 2 | 4 | 4 | 1 | 3 | (11) | 9316.7719 | 832.3 | –1.5 |
| 5 | 2 | 4 | 4 | 1 | 3 | (12) | 10392.8889 | –140.0 | 6.1 |
| 5 | 2 | 4 | 4 | 2 | 3 | (00) | 8581.5276 | 164.9 | –0.2 |
| 5 | 2 | 4 | 4 | 2 | 3 | (10) | 8474.0464 | –174.3 | 0.8 |
| 5 | 2 | 4 | 4 | 2 | 3 | (01) | 8504.7908 | –208.2 | 2.1 |
| 5 | 2 | 4 | 4 | 2 | 3 | (11) | 8687.2181 | –225.1 | –1.3 |
| 5 | 2 | 4 | 4 | 2 | 3 | (12) | 8199.4987 | 286.8 | 4.3 |
| **5** | **3** | **2** | **4** | **2** | **2** | **(01)** | **13928.7698** | **661.2** | **4.3** |
| 5 | 3 | 2 | 4 | 2 | 3 | (00) | 14622.2988 | 284.0 | –12.1 |
| 5 | 3 | 2 | 4 | 2 | 3 | (10) | 15447.6587 | 305.9 | –6.5 |
| 5 | 3 | 2 | 4 | 3 | 1 | (00) | 9703.3841 | 388.6 | –5.7 |
| 5 | 3 | 2 | 4 | 3 | 1 | (01) | 9517.9445 | –153.3 | –0.1 |
| **5** | **3** | **2** | **4** | **3** | **2** | **(01)** | **12348.3831** | **1487.8** | **1.3** |
| 5 | 3 | 3 | 4 | 2 | 2 | (00) | 12247.7071 | –165.9 | –8.5 |
| 5 | 3 | 3 | 4 | 2 | 2 | (10) | 11845.7344 | 49.6 | 3.4 |
| 5 | 3 | 3 | 4 | 2 | 2 | (01) | 11173.1201 | –711.6 | 7.8 |
| 5 | 3 | 3 | 4 | 2 | 2 | (11) | 11180.2527 | 657.0 | –11.4 |
| 5 | 3 | 3 | 4 | 2 | 2 | (12) | 11351.1927 | 563.2 | 25.4 |
| **5** | **3** | **3** | **4** | **2** | **3** | **(11)** | **13224.4367** | **528.4** | **–5.3** |
| 5 | 3 | 3 | 4 | 3 | 2 | (00) | 9160.3210 | 253.3 | –0.3 |
| 5 | 3 | 3 | 4 | 3 | 2 | (10) | 9252.7224 | –365.3 | 6.4 |
| 5 | 3 | 3 | 4 | 3 | 2 | (01) | 9592.7301 | 111.8 | 1.4 |
| 5 | 3 | 3 | 4 | 3 | 2 | (11) | 9413.0454 | 316.4 | –7.6 |
| 5 | 3 | 3 | 4 | 3 | 2 | (12) | 9330.2848 | 200.3 | –1.0 |
| 5 | 4 | 1 | 4 | 3 | 2 | (00) | 15090.2571 | –65.4 | 2.0 |
| 5 | 4 | 1 | 4 | 4 | 0 | (00) | 9288.3103 | 306.5 | –1.8 |
| 5 | 4 | 2 | 4 | 3 | 1 | (00) | 14802.7433 | –166.1 | 2.5 |
| 6 | 0 | 6 | 5 | 0 | 5 | (00) | 8844.6952 | 91.8 | –0.4 |
| 6 | 0 | 6 | 5 | 0 | 5 | (10) | 8860.3317 | 18.6 | –1.8 |
| 6 | 0 | 6 | 5 | 0 | 5 | (01) | 8866.9624 | 7.0 | –1.2 |
| 6 | 0 | 6 | 5 | 0 | 5 | (11) | 8838.4529 | 34.9 | 0.3 |
| 6 | 0 | 6 | 5 | 0 | 5 | (12) | 8931.5488 | –110.1 | 2.0 |
| 6 | 0 | 6 | 5 | 1 | 5 | (00) | 8825.4940 | 103.1 | 0.6 |
| 6 | 0 | 6 | 5 | 1 | 5 | (10) | 8809.9819 | –23.6 | –3.7 |
| 6 | 0 | 6 | 5 | 1 | 5 | (01) | 8809.7683 | –21.1 | –0.1 |
| 6 | 0 | 6 | 5 | 1 | 5 | (11) | 8822.8652 | 2.8 | 0.0 |
| 6 | 0 | 6 | 5 | 1 | 5 | (12) | 8741.7793 | 62.9 | –2.4 |

| | | | | | | | | | |
|---|---|---|---|---|---|---|---|---|---|
| 6 | 1 | 5 | 5 | 1 | 4 | (00) | 10324.6764 | 88.9 | –1.0 |
| 6 | 1 | 5 | 5 | 1 | 4 | (10) | 10442.4792 | 19.0 | 0.3 |
| 6 | 1 | 5 | 5 | 1 | 4 | (01) | 10466.0718 | –18.7 | 0.9 |
| 6 | 1 | 5 | 5 | 1 | 4 | (11) | 10293.9815 | 282.7 | –0.5 |
| 6 | 1 | 5 | 5 | 1 | 4 | (12) | 10421.0853 | 262.1 | 1.7 |
| 6 | 1 | 5 | 5 | 2 | 4 | (00) | 9891.1927 | 249.0 | –0.2 |
| 6 | 1 | 5 | 5 | 2 | 4 | (10) | 9565.2644 | –487.5 | –4.7 |
| 6 | 1 | 5 | 5 | 2 | 4 | (01) | 9527.9710 | –331.0 | 7.0 |
| 6 | 1 | 5 | 5 | 2 | 4 | (11) | 9957.6787 | –347.9 | 0.9 |
| 6 | 1 | 5 | 5 | 2 | 4 | (12) | 8593.7341 | 899.3 | –4.0 |
| 6 | 1 | 6 | 5 | 0 | 5 | (00) | 8850.5104 | 87.9 | –0.4 |
| 6 | 1 | 6 | 5 | 0 | 5 | (10) | 8876.1884 | 34.1 | –1.1 |
| 6 | 1 | 6 | 5 | 0 | 5 | (01) | 8884.8034 | 14.7 | –1.5 |
| 6 | 1 | 6 | 5 | 0 | 5 | (11) | 8843.1849 | 43.4 | –0.2 |
| 6 | 1 | 6 | 5 | 0 | 5 | (12) | 8997.0173 | –179.1 | 2.5 |
| 6 | 1 | 6 | 5 | 1 | 5 | (00) | 8831.3084 | 98.4 | –0.2 |
| 6 | 1 | 6 | 5 | 1 | 5 | (10) | 8825.8391 | –7.6 | –2.5 |
| 6 | 1 | 6 | 5 | 1 | 5 | (01) | 8827.6102 | –12.5 | 0.5 |
| 6 | 1 | 6 | 5 | 1 | 5 | (11) | 8827.6096 | 23.7 | 11.9 |
| 6 | 1 | 6 | 5 | 1 | 5 | (12) | 8807.2493 | –4.5 | –0.4 |
| 6 | 2 | 4 | 5 | 1 | 5 | (10) | 20055.1954 | –809.0 | 13.1 |
| 6 | 2 | 4 | 5 | 1 | 5 | (11) | 20453.2941 | –283.0 | –17.4 |
| 6 | 2 | 4 | 5 | 1 | 5 | (12) | 19537.3193 | 625.9 | –1.3 |
| 6 | 2 | 4 | 5 | 2 | 3 | (00) | 11709.8169 | 248.8 | –1.2 |
| 6 | 2 | 4 | 5 | 2 | 3 | (10) | 11157.9480 | –644.3 | 3.8 |
| 6 | 2 | 4 | 5 | 2 | 3 | (01) | 11010.9436 | –530.3 | 2.0 |
| 6 | 2 | 4 | 5 | 2 | 3 | (11) | 11587.8926 | –371.2 | –4.8 |
| 6 | 2 | 4 | 5 | 2 | 3 | (12) | 10343.3838 | 576.9 | 11.3 |
| 6 | 2 | 4 | 5 | 3 | 3 | (00) | 9401.9190 | 693.6 | –9.1 |
| 6 | 2 | 4 | 5 | 3 | 3 | (10) | 8679.6734 | –1157.1 | 8.6 |
| 6 | 2 | 4 | 5 | 3 | 3 | (01) | 8965.6923 | –550.8 | –5.7 |
| 6 | 2 | 4 | 5 | 3 | 3 | (11) | 10154.2069 | –1380.9 | –4.9 |
| 6 | 2 | 4 | 5 | 3 | 3 | (12) | 7569.1691 | 534.8 | 10.7 |
| 6 | 2 | 5 | 5 | 1 | 4 | (00) | 10505.3169 | –1.2 | –0.9 |
| 6 | 2 | 5 | 5 | 1 | 4 | (10) | 10872.5840 | 377.9 | 5.1 |
| 6 | 2 | 5 | 5 | 1 | 4 | (01) | 10939.3701 | 157.1 | –4.6 |
| 6 | 2 | 5 | 5 | 1 | 4 | (11) | 10437.4388 | 546.3 | –2.1 |
| 6 | 2 | 5 | 5 | 1 | 4 | (12) | 11613.9477 | –385.7 | 9.0 |
| 6 | 2 | 5 | 5 | 2 | 4 | (00) | 10071.8331 | 158.8 | –0.2 |
| 6 | 2 | 5 | 5 | 2 | 4 | (10) | 9995.3687 | –129.1 | –0.3 |
| 6 | 2 | 5 | 5 | 2 | 4 | (01) | 10001.2689 | –155.5 | 1.1 |
| 6 | 2 | 5 | 5 | 2 | 4 | (11) | 10101.1363 | –84.1 | –0.4 |
| 6 | 2 | 5 | 5 | 2 | 4 | (12) | 9786.5963 | 251.4 | 3.1 |
| 6 | 3 | 3 | 5 | 2 | 4 | (00) | 17957.1366 | 568.6 | –18.5 |

| | | | | | | | | | |
|---|---|---|---|---|---|---|---|---|---|
| 6 | 3 | 3 | 5 | 2 | 4 | (10) | 18687.1542 | 660.7 | –0.1 |
| 6 | 3 | 3 | 5 | 3 | 2 | (00) | 11916.3691 | 453.1 | –2.9 |
| 6 | 3 | 3 | 5 | 3 | 2 | (10) | 11713.5416 | 180.1 | 6.9 |
| 6 | 3 | 3 | 5 | 3 | 2 | (01) | 11372.5177 | –869.4 | –4.3 |
| **6** | **3** | **3** | **5** | **3** | **3** | **(10)** | **13278.2850** | **192.3** | **–6.7** |
| 6 | 3 | 4 | 5 | 2 | 3 | (00) | 13205.5478 | –200.1 | –11.3 |
| 6 | 3 | 4 | 5 | 2 | 3 | (10) | 13298.2486 | 146.1 | 4.7 |
| 6 | 3 | 4 | 5 | 2 | 3 | (01) | 13072.1858 | –246.4 | 3.3 |
| 6 | 3 | 4 | 5 | 2 | 3 | (11) | 12630.6880 | 975.8 | –5.7 |
| 6 | 3 | 4 | 5 | 2 | 3 | (12) | 13360.0388 | 321.3 | 5.7 |
| 6 | 3 | 4 | 5 | 3 | 3 | (00) | 10897.6677 | 262.5 | –1.4 |
| 6 | 3 | 4 | 5 | 3 | 3 | (10) | 10819.9679 | –372.7 | 3.4 |
| 6 | 3 | 4 | 5 | 3 | 3 | (01) | 11026.9346 | –266.8 | –4.3 |
| 6 | 3 | 4 | 5 | 3 | 3 | (11) | 11197.0026 | –33.5 | –5.5 |
| 6 | 3 | 4 | 5 | 3 | 3 | (12) | 10585.8282 | 283.2 | 9.3 |
| 6 | 4 | 2 | 5 | 3 | 3 | (00) | 17273.2111 | 102.9 | 0.3 |
| 6 | 4 | 2 | 5 | 4 | 1 | (00) | 11343.2760 | 422.6 | –1.0 |
| 6 | 4 | 3 | 5 | 3 | 2 | (00) | 16234.9867 | –219.7 | 8.3 |
| 7 | 0 | 7 | 6 | 0 | 6 | (00) | 10200.4208 | 104.9 | 0.1 |
| 7 | 0 | 7 | 6 | 0 | 6 | (10) | 10205.6763 | 8.1 | –4.0 |
| 7 | 0 | 7 | 6 | 0 | 6 | (01) | 10208.7443 | –2.6 | –1.0 |
| 7 | 0 | 7 | 6 | 0 | 6 | (11) | 10193.8378 | 21.9 | 0.6 |
| 7 | 0 | 7 | 6 | 0 | 6 | (12) | 10237.8442 | –88.6 | 0.9 |
| 7 | 0 | 7 | 6 | 1 | 6 | (00) | 10194.6054 | 108.6 | –0.1 |
| 7 | 0 | 7 | 6 | 1 | 6 | (10) | 10189.8215 | –5.5 | –2.8 |
| 7 | 0 | 7 | 6 | 1 | 6 | (01) | 10190.9032 | –10.3 | –0.8 |
| 7 | 0 | 7 | 6 | 1 | 6 | (11) | 10189.1051 | 12.7 | 0.4 |
| 7 | 0 | 7 | 6 | 1 | 6 | (12) | 10172.3741 | –21.2 | –1.1 |
| 7 | 1 | 6 | 6 | 1 | 5 | (00) | 11613.1492 | 101.9 | –1.2 |
| 7 | 1 | 6 | 6 | 1 | 5 | (10) | 11716.7665 | 89.2 | 0.6 |
| 7 | 1 | 6 | 6 | 1 | 5 | (01) | 11744.5645 | –3.3 | –0.5 |
| 7 | 1 | 6 | 6 | 1 | 5 | (11) | 11587.0427 | 183.8 | –0.7 |
| 7 | 1 | 6 | 6 | 1 | 5 | (12) | 11932.9636 | –38.8 | 2.5 |
| 7 | 1 | 6 | 6 | 2 | 5 | (00) | 11432.5099 | 193.2 | –0.1 |
| 7 | 1 | 6 | 6 | 2 | 5 | (10) | 11286.6621 | –269.3 | –3.8 |
| 7 | 1 | 6 | 6 | 2 | 5 | (01) | 11271.2672 | –178.1 | 6.0 |
| 7 | 1 | 6 | 6 | 2 | 5 | (11) | 11443.5853 | –79.9 | 0.8 |
| 7 | 1 | 6 | 6 | 2 | 5 | (12) | 10740.1024 | 610.2 | –3.6 |
| 7 | 1 | 7 | 6 | 0 | 6 | (00) | 10202.1028 | 103.3 | –0.2 |
| 7 | 1 | 7 | 6 | 0 | 6 | (10) | 10210.3975 | 14.2 | –3.1 |
| 7 | 1 | 7 | 6 | 0 | 6 | (01) | 10213.9960 | –0.4 | –0.8 |
| 7 | 1 | 7 | 6 | 0 | 6 | (11) | 10195.2118 | 24.2 | 0.6 |
| 7 | 1 | 7 | 6 | 0 | 6 | (12) | 10258.4170 | –111.7 | 0.8 |
| 7 | 1 | 7 | 6 | 1 | 6 | (00) | 10196.2875 | 107.1 | –0.2 |

| | | | | | | | | | |
|---|---|---|---|---|---|---|---|---|---|
| 7 | 1 | 7 | 6 | 1 | 6 | (10) | 10194.5409 | –1.2 | –3.6 |
| 7 | 1 | 7 | 6 | 1 | 6 | (01) | 10196.1551 | –8.0 | –0.4 |
| 7 | 1 | 7 | 6 | 1 | 6 | (11) | 10190.4797 | 15.6 | 0.9 |
| 7 | 1 | 7 | 6 | 1 | 6 | (12) | 10192.9476 | –43.7 | –0.5 |
| 7 | 2 | 5 | 6 | 2 | 4 | (00) | 13218.6926 | 159.9 | 0.0 |
| 7 | 2 | 5 | 6 | 2 | 4 | (10) | 13023.5994 | –493.6 | 2.6 |
| 7 | 2 | 5 | 6 | 2 | 4 | (01) | 12962.8905 | –317.6 | 5.3 |
| 7 | 2 | 5 | 6 | 2 | 4 | (11) | 13136.5278 | 166.3 | –3.2 |
| 7 | 2 | 5 | 6 | 2 | 4 | (12) | 12335.7745 | 649.5 | 4.0 |
| 7 | 2 | 5 | 6 | 3 | 4 | (00) | 11722.9487 | 595.8 | –3.0 |
| 7 | 2 | 5 | 6 | 3 | 4 | (10) | 10883.2980 | –1284.9 | 0.9 |
| 7 | 2 | 5 | 6 | 3 | 4 | (01) | 10901.6464 | –603.4 | 2.1 |
| 7 | 2 | 5 | 6 | 3 | 4 | (11) | 12093.7332 | –1179.9 | –1.6 |
| 7 | 2 | 5 | 6 | 3 | 4 | (12) | 9319.1201 | 905.8 | 10.2 |
| 7 | 2 | 6 | 6 | 1 | 5 | (00) | 11679.6374 | 60.5 | –0.7 |
| 7 | 2 | 6 | 6 | 1 | 5 | (10) | 11889.4036 | 273.4 | 3.8 |
| 7 | 2 | 6 | 6 | 1 | 5 | (01) | 11935.7722 | 60.7 | –4.3 |
| 7 | 2 | 6 | 6 | 1 | 5 | (11) | 11640.3644 | 273.1 | –2.3 |
| 7 | 2 | 6 | 6 | 1 | 5 | (12) | 12533.3598 | –474.6 | 9.1 |
| 7 | 2 | 6 | 6 | 2 | 5 | (00) | 11498.9974 | 151.1 | –0.3 |
| 7 | 2 | 6 | 6 | 2 | 5 | (10) | 11459.2988 | –85.5 | –1.0 |
| 7 | 2 | 6 | 6 | 2 | 5 | (01) | 11462.4743 | –114.7 | 1.6 |
| 7 | 2 | 6 | 6 | 2 | 5 | (11) | 11496.9076 | 10.0 | –0.2 |
| 7 | 2 | 6 | 6 | 2 | 5 | (12) | 11340.4976 | 173.3 | 2.0 |
| 7 | 3 | 4 | 6 | 2 | 5 | (10) | 22049.8088 | 158.7 | 12.8 |
| 7 | 3 | 4 | 6 | 3 | 3 | (00) | 14006.4677 | 435.7 | –3.6 |
| 7 | 3 | 4 | 6 | 3 | 3 | (10) | 13358.0248 | –629.5 | 14.1 |
| 7 | 3 | 4 | 6 | 3 | 3 | (01) | 13031.8511 | –956.4 | –5.9 |
| 7 | 3 | 5 | 6 | 2 | 4 | (00) | 14034.0747 | –194.0 | –7.6 |
| 7 | 3 | 5 | 6 | 2 | 4 | (10) | 14543.8228 | 501.7 | 4.2 |
| 7 | 3 | 5 | 6 | 2 | 4 | (01) | 14533.7183 | –4.8 | 1.1 |
| 7 | 3 | 5 | 6 | 2 | 4 | (11) | 13753.3556 | 1098.6 | –4.1 |
| 7 | 3 | 5 | 6 | 2 | 4 | (12) | 15098.0228 | 90.1 | 2.6 |
| 7 | 3 | 5 | 6 | 3 | 4 | (00) | 12538.3403 | 251.4 | –1.1 |
| 7 | 3 | 5 | 6 | 3 | 4 | (10) | 12403.5233 | –287.7 | 4.4 |
| 7 | 3 | 5 | 6 | 3 | 4 | (01) | 12472.4742 | –290.5 | –2.1 |
| 7 | 3 | 5 | 6 | 3 | 4 | (11) | 12710.5603 | –248.3 | –3.2 |
| 7 | 3 | 5 | 6 | 3 | 4 | (12) | 12081.3663 | 344.3 | 6.7 |
| 7 | 4 | 3 | 6 | 3 | 4 | (00) | 19929.2168 | 392.9 | –4.1 |
| 7 | 4 | 4 | 6 | 3 | 3 | (00) | 17322.4585 | –333.0 | –12.1 |
| 7 | 4 | 4 | 6 | 4 | 3 | (00) | 13003.8616 | 360.6 | –2.6 |
| 8 | 0 | 8 | 7 | 0 | 7 | (00) | 11559.6508 | 114.9 | –0.1 |
| 8 | 0 | 8 | 7 | 0 | 7 | (10) | 11560.7079 | 6.1 | –3.2 |
| 8 | 0 | 8 | 7 | 0 | 7 | (01) | 11562.1865 | –6.5 | –1.0 |

| | | | | | | | | | |
|---|---|---|---|---|---|---|---|---|---|
| 8 | 0 | 8 | 7 | 0 | 7 | (12) | 11574.8680 | –85.0 | –0.7 |
| 8 | 0 | 8 | 7 | 1 | 7 | (00) | 11557.9637 | 111.4 | –5.0 |
| 8 | 0 | 8 | 7 | 1 | 7 | (10) | 11555.9877 | 1.1 | –3.1 |
| 8 | 0 | 8 | 7 | 1 | 7 | (01) | 11556.9355 | –7.9 | –0.5 |
| 8 | 0 | 8 | 7 | 1 | 7 | (11) | 11551.2532 | 13.1 | 1.2 |
| 8 | 0 | 8 | 7 | 1 | 7 | (12) | 11554.2946 | –62.5 | –1.2 |
| 8 | 1 | 7 | 7 | 1 | 6 | (00) | 12932.2489 | 121.9 | –0.9 |
| 8 | 1 | 7 | 7 | 1 | 6 | (10) | 12982.9984 | 55.5 | 0.3 |
| 8 | 1 | 7 | 7 | 1 | 6 | (01) | 12998.8663 | –50.8 | –1.4 |
| 8 | 1 | 7 | 7 | 1 | 6 | (11) | 12908.7673 | 113.5 | –0.2 |
| 8 | 1 | 7 | 7 | 1 | 6 | (12) | 13180.9205 | –142.7 | 4.2 |
| 8 | 1 | 7 | 7 | 2 | 6 | (00) | 12865.7620 | 164.6 | 0.0 |
| 8 | 1 | 7 | 7 | 2 | 6 | (10) | 12810.3618 | –128.2 | –2.4 |
| 8 | 1 | 7 | 7 | 2 | 6 | (01) | 12807.6598 | –113.6 | 3.5 |
| 8 | 1 | 7 | 7 | 2 | 6 | (11) | 12855.4450 | 23.6 | 0.8 |
| 8 | 1 | 7 | 7 | 2 | 6 | (12) | 12580.5251 | 293.9 | –1.6 |
| 8 | 1 | 8 | 7 | 0 | 7 | (00) | 11560.1210 | 113.8 | –0.8 |
| 8 | 1 | 8 | 7 | 0 | 7 | (10) | 11562.0607 | 8.3 | –2.7 |
| 8 | 1 | 8 | 7 | 0 | 7 | (01) | 11563.6736 | –6.0 | –0.8 |
| 8 | 1 | 8 | 7 | 0 | 7 | (11) | 11553.0134 | 15.7 | 1.0 |
| 8 | 1 | 8 | 7 | 0 | 7 | (12) | 11580.9806 | –91.2 | 0.1 |
| 8 | 1 | 8 | 7 | 1 | 7 | (00) | 11558.4404 | 116.8 | 0.8 |
| 8 | 1 | 8 | 7 | 1 | 7 | (10) | 11557.3399 | 2.7 | –3.3 |
| 8 | 1 | 8 | 7 | 1 | 7 | (01) | 11558.4211 | –8.9 | –1.9 |
| 8 | 1 | 8 | 7 | 1 | 7 | (11) | 11551.6399 | 13.9 | 1.5 |
| 8 | 1 | 8 | 7 | 1 | 7 | (12) | 11560.4070 | –68.9 | –0.7 |
| 8 | 2 | 6 | 7 | 2 | 5 | (00) | 14520.9427 | 94.5 | –0.8 |
| 8 | 2 | 6 | 7 | 2 | 5 | (10) | 14633.7302 | –107.3 | 0.5 |
| 8 | 2 | 6 | 7 | 2 | 5 | (01) | 14641.3923 | –130.2 | 3.2 |
| 8 | 2 | 6 | 7 | 2 | 5 | (11) | 14458.1276 | 391.3 | –3.6 |
| 8 | 2 | 6 | 7 | 2 | 5 | (12) | 14378.6829 | 477.6 | 0.2 |
| 8 | 2 | 6 | 7 | 3 | 5 | (00) | 13705.5524 | 440.2 | –1.4 |
| 8 | 2 | 6 | 7 | 3 | 5 | (10) | 13113.5065 | –1102.8 | –1.4 |
| 8 | 2 | 6 | 7 | 3 | 5 | (01) | 13070.5654 | –442.2 | 8.2 |
| 8 | 2 | 6 | 7 | 3 | 5 | (11) | 13841.3024 | –538.4 | –0.1 |
| 8 | 2 | 6 | 7 | 3 | 5 | (12) | 11616.4376 | 1040.0 | 4.6 |
| 8 | 2 | 7 | 7 | 1 | 6 | (00) | 12954.8046 | 104.8 | –0.8 |
| 8 | 2 | 7 | 7 | 1 | 6 | (10) | 13044.4533 | 133.5 | 1.4 |
| 8 | 2 | 7 | 7 | 1 | 6 | (01) | 13066.6167 | –34.1 | –3.4 |
| 8 | 2 | 7 | 7 | 1 | 6 | (11) | 12926.9824 | 141.0 | –0.2 |
| 8 | 2 | 7 | 7 | 1 | 6 | (12) | 13424.6360 | –344.7 | 7.6 |
| 8 | 2 | 7 | 7 | 2 | 6 | (00) | 12888.3175 | 147.3 | –0.2 |
| 8 | 2 | 7 | 7 | 2 | 6 | (10) | 12871.8170 | –49.9 | –1.0 |
| 8 | 2 | 7 | 7 | 2 | 6 | (01) | 12875.4099 | –97.2 | 1.3 |

| | | | | | | | | | |
|---|---|---|---|---|---|---|---|---|---|
| 8 | 2 | 7 | 7 | 2 | 6 | (11) | 12873.6603 | 51.3 | 1.0 |
| 8 | 2 | 7 | 7 | 2 | 6 | (12) | 12824.2406 | 91.9 | 1.8 |
| 8 | 3 | 5 | 7 | 3 | 4 | (10) | 15101.2219 | –1055.6 | –4.7 |
| 8 | 3 | 5 | 7 | 3 | 4 | (01) | 14890.5475 | –674.9 | –2.5 |
| 8 | 3 | 6 | 7 | 2 | 5 | (00) | 14896.4364 | –118.7 | –0.1 |
| 8 | 3 | 6 | 7 | 2 | 5 | (10) | 15487.0312 | 758.4 | 3.8 |
| 8 | 3 | 6 | 7 | 2 | 5 | (01) | 15554.9739 | 65.7 | –2.9 |
| 8 | 3 | 6 | 7 | 2 | 5 | (11) | 14752.4588 | 819.6 | –6.2 |
| 8 | 3 | 6 | 7 | 2 | 5 | (12) | 16445.0308 | –231.9 | 1.5 |
| 8 | 3 | 6 | 7 | 3 | 5 | (00) | 14081.0464 | 227.3 | –0.4 |
| 8 | 3 | 6 | 7 | 3 | 5 | (10) | 13966.8073 | –237.4 | 1.8 |
| 8 | 3 | 6 | 7 | 3 | 5 | (01) | 13984.1443 | –249.0 | –0.5 |
| 8 | 3 | 6 | 7 | 3 | 5 | (11) | 14135.6332 | –110.5 | –3.1 |
| 8 | 3 | 6 | 7 | 3 | 5 | (12) | 13682.7842 | 329.2 | 4.6 |
| 8 | 4 | 5 | 7 | 3 | 4 | (00) | 18117.5174 | –402.3 | –4.0 |
| 9 | 0 | 9 | 8 | 0 | 8 | (00) | 12920.0683 | 116.0 | –7.5 |
| 9 | 0 | 9 | 8 | 0 | 8 | (10) | 12919.3337 | 3.3 | –5.1 |
| 9 | 0 | 9 | 8 | 0 | 8 | (01) | 12920.0005 | –9.6 | –1.2 |
| 9 | 0 | 9 | 8 | 0 | 8 | (12) | 12925.8000 | –93.1 | –0.8 |
| 9 | 0 | 9 | 8 | 1 | 8 | (00) | 12919.6050 | 124.0 | 0.1 |
| 9 | 0 | 9 | 8 | 1 | 8 | (10) | 12917.9828 | 3.0 | –3.7 |
| 9 | 0 | 9 | 8 | 1 | 8 | (01) | 12918.5139 | –9.6 | –0.8 |
| 9 | 0 | 9 | 8 | 1 | 8 | (12) | 12919.6914 | –82.8 | 2.5 |
| 9 | 1 | 8 | 8 | 1 | 7 | (00) | 14274.6249 | 135.8 | –0.8 |
| 9 | 1 | 8 | 8 | 1 | 7 | (10) | 14293.7076 | 18.8 | –2.3 |
| 9 | 1 | 8 | 8 | 1 | 7 | (01) | 14301.3310 | –81.6 | 0.7 |
| 9 | 1 | 8 | 8 | 1 | 7 | (11) | 14251.6720 | 83.3 | 0.9 |
| 9 | 1 | 8 | 8 | 1 | 7 | (12) | 14401.1157 | –97.2 | 2.3 |
| 9 | 1 | 8 | 8 | 2 | 7 | (00) | 14252.0700 | 153.6 | –0.1 |
| 9 | 1 | 8 | 8 | 2 | 7 | (10) | 14232.2541 | –57.8 | –2.0 |
| 9 | 1 | 8 | 8 | 2 | 7 | (01) | 14233.5800 | –98.9 | 2.1 |
| 9 | 1 | 8 | 8 | 2 | 7 | (11) | 14233.4568 | 55.8 | 0.8 |
| 9 | 1 | 8 | 8 | 2 | 7 | (12) | 14157.4016 | 106.1 | 0.3 |
| 9 | 1 | 9 | 8 | 0 | 8 | (00) | 12920.2040 | 123.0 | –0.3 |
| 9 | 1 | 9 | 8 | 0 | 8 | (10) | 12919.7116 | 5.0 | –3.9 |
| 9 | 1 | 9 | 8 | 0 | 8 | (01) | 12920.4103 | –9.1 | –0.7 |
| 9 | 1 | 9 | 8 | 0 | 8 | (11) | 12912.7829 | 10.0 | 1.4 |
| 9 | 1 | 9 | 8 | 0 | 8 | (12) | 12927.5485 | –94.6 | –0.4 |
| 9 | 1 | 9 | 8 | 1 | 8 | (10) | 12918.3607 | 4.8 | –2.5 |
| 9 | 1 | 9 | 8 | 1 | 8 | (01) | 12918.9233 | –9.6 | –0.8 |
| 9 | 1 | 9 | 8 | 1 | 8 | (12) | 12921.4365 | –87.8 | –0.6 |
| 9 | 2 | 7 | 8 | 2 | 6 | (00) | 15766.6294 | 94.3 | –0.6 |
| 9 | 2 | 7 | 8 | 2 | 6 | (10) | 15938.9906 | 164.6 | –0.4 |
| 9 | 2 | 7 | 8 | 2 | 6 | (01) | 15971.6695 | –86.0 | 0.4 |

| | | | | | | | | | |
|---|---|---|---|---|---|---|---|---|---|
| 9 | 2 | 7 | 8 | 2 | 6 | (11) | 15715.0367 | 299.9 | –3.9 |
| 9 | 2 | 7 | 8 | 2 | 6 | (12) | 16127.9109 | 127.7 | 1.0 |
| 9 | 2 | 7 | 8 | 3 | 6 | (10) | 15085.6902 | –700.5 | –3.1 |
| 9 | 2 | 7 | 8 | 3 | 6 | (01) | 15058.0903 | –279.5 | 8.9 |
| 9 | 2 | 7 | 8 | 3 | 6 | (11) | 15420.7037 | –130.2 | –3.2 |
| 9 | 2 | 7 | 8 | 3 | 6 | (12) | 14061.5611 | 835.3 | –2.1 |
| 9 | 2 | 8 | 8 | 1 | 7 | (00) | 14281.8563 | 130.4 | 0.2 |
| 9 | 2 | 8 | 8 | 1 | 7 | (10) | 14314.0542 | 51.0 | 0.2 |
| 9 | 2 | 8 | 8 | 1 | 7 | (01) | 14323.5660 | –79.8 | –1.2 |
| 9 | 2 | 8 | 8 | 1 | 7 | (12) | 14487.7917 | –170.3 | 4.3 |
| 9 | 2 | 8 | 8 | 2 | 7 | (00) | 14259.2999 | 146.7 | –0.6 |
| 9 | 2 | 8 | 8 | 2 | 7 | (10) | 14252.5989 | –27.5 | –1.3 |
| 9 | 2 | 8 | 8 | 2 | 7 | (01) | 14255.8161 | –96.0 | 1.3 |
| 9 | 2 | 8 | 8 | 2 | 7 | (12) | 14244.0777 | 33.1 | 2.4 |
| 9 | 3 | 6 | 8 | 3 | 5 | (10) | 17023.4677 | –979.2 | 9.9 |
| 9 | 3 | 6 | 8 | 3 | 5 | (01) | 16916.3232 | –445.6 | 1.6 |
| 9 | 3 | 7 | 8 | 2 | 6 | (00) | 15918.5237 | –11.1 | 2.0 |
| 9 | 3 | 7 | 8 | 2 | 6 | (10) | 16326.2927 | 692.4 | 3.6 |
| 9 | 3 | 7 | 8 | 2 | 6 | (01) | 16391.8722 | –18.7 | –6.2 |
| 9 | 3 | 7 | 8 | 2 | 6 | (11) | 15835.9450 | 454.8 | –3.1 |
| 9 | 3 | 7 | 8 | 2 | 6 | (12) | 17348.8984 | –450.9 | 2.0 |
| 9 | 3 | 7 | 8 | 3 | 6 | (00) | 15543.0284 | 200.4 | –0.4 |
| 9 | 3 | 7 | 8 | 3 | 6 | (10) | 15472.9923 | –172.7 | 0.9 |
| 9 | 3 | 7 | 8 | 3 | 6 | (01) | 15478.2919 | –213.3 | 1.2 |
| 9 | 3 | 7 | 8 | 3 | 6 | (11) | 15541.6132 | 25.8 | –1.1 |
| 9 | 3 | 7 | 8 | 3 | 6 | (12) | 15282.5524 | 260.5 | 2.6 |
| 9 | 4 | 6 | 8 | 3 | 5 | (00) | 18753.3278 | –417.2 | –0.8 |
| 10 | 0 | 10 | 9 | 0 | 9 | (01) | 14279.4496 | –15.7 | –3.6 |
| 10 | 0 | 10 | 9 | 1 | 9 | (01) | 14279.0424 | –13.5 | –1.4 |
| 10 | 1 | 9 | 9 | 1 | 8 | (00) | 15627.9258 | 145.2 | 0.6 |
| 10 | 1 | 9 | 9 | 1 | 8 | (10) | 15632.5431 | 3.4 | –1.8 |
| 10 | 1 | 9 | 9 | 1 | 8 | (01) | 15636.1242 | –100.7 | 0.3 |
| 10 | 1 | 9 | 9 | 2 | 8 | (00) | 15620.6951 | 151.3 | 0.3 |
| 10 | 1 | 9 | 9 | 2 | 8 | (10) | 15612.1990 | –26.2 | –1.8 |
| 10 | 1 | 9 | 9 | 2 | 8 | (01) | 15613.8884 | –103.3 | 1.4 |
| 10 | 1 | 9 | 9 | 2 | 8 | (12) | 15595.8911 | 21.6 | 2.1 |
| 10 | 1 | 10 | 9 | 0 | 9 | (00) | 14280.9349 | 130.5 | 0.4 |
| 10 | 1 | 10 | 9 | 0 | 9 | (01) | 14279.5622 | –13.4 | –1.3 |
| 10 | 1 | 10 | 9 | 1 | 9 | (10) | 14279.0424 | 40.6 | 33.5 |
| 10 | 1 | 10 | 9 | 1 | 9 | (01) | 14279.1633 | –3.0 | 9.2 |
| 10 | 1 | 10 | 9 | 1 | 9 | (11) | 14273.1140 | –24.3 | –26.7 |
| 10 | 2 | 8 | 9 | 2 | 7 | (10) | 17151.7163 | 168.3 | 0.9 |
| 10 | 2 | 8 | 9 | 2 | 7 | (01) | 17175.5675 | –144.4 | –2.6 |
| 10 | 2 | 8 | 9 | 2 | 7 | (12) | 17469.4403 | –92.7 | 0.6 |

| | | | | | | | | | |
|---|---|---|---|---|---|---|---|---|---|
| 10 | 2 | 8 | 9 | 3 | 7 | (10) | 16764.4142 | –359.6 | –3.2 |
| 10 | 2 | 8 | 9 | 3 | 7 | (01) | 16755.3666 | –209.9 | 5.9 |
| 10 | 2 | 8 | 9 | 3 | 7 | (11) | 16881.1782 | 46.3 | –0.2 |
| 10 | 2 | 8 | 9 | 3 | 7 | (12) | 16248.4542 | 487.3 | 1.0 |
| 10 | 2 | 9 | 9 | 1 | 8 | (00) | 15630.1484 | 143.0 | 0.6 |
| 10 | 2 | 9 | 9 | 1 | 8 | (10) | 15638.9550 | 15.2 | –0.7 |
| 10 | 2 | 9 | 9 | 1 | 8 | (01) | 15643.0613 | –100.9 | –0.3 |
| 10 | 2 | 9 | 9 | 1 | 8 | (12) | 15711.1089 | –76.5 | 3.0 |
| 10 | 2 | 9 | 9 | 2 | 8 | (00) | 15622.9171 | 148.5 | –0.3 |
| 10 | 2 | 9 | 9 | 2 | 8 | (10) | 15618.6102 | –15.1 | –1.4 |
| 10 | 2 | 9 | 9 | 2 | 8 | (01) | 15620.8280 | –101.0 | 3.4 |
| 10 | 2 | 9 | 9 | 2 | 8 | (12) | 15624.4340 | –2.3 | 2.1 |
| 10 | 3 | 7 | 9 | 3 | 6 | (10) | 18785.0305 | –420.4 | 2.1 |
| 10 | 3 | 7 | 9 | 3 | 6 | (01) | 18764.3742 | –274.0 | –7.1 |
| 10 | 3 | 8 | 9 | 2 | 7 | (00) | 17102.8674 | 71.3 | –0.3 |
| 10 | 3 | 8 | 9 | 2 | 7 | (10) | 17304.0301 | 420.9 | 3.3 |
| 10 | 3 | 8 | 9 | 2 | 7 | (01) | 17340.6912 | –135.9 | –7.1 |
| 10 | 3 | 8 | 9 | 2 | 7 | (11) | 17047.2325 | 243.8 | –2.6 |
| 10 | 3 | 8 | 9 | 2 | 7 | (12) | 18042.3040 | –398.6 | 5.6 |
| 10 | 3 | 8 | 9 | 3 | 7 | (00) | 16950.9753 | 178.9 | –0.7 |
| 10 | 3 | 8 | 9 | 3 | 7 | (10) | 16916.7285 | –106.4 | –0.2 |
| 10 | 3 | 8 | 9 | 3 | 7 | (01) | 16920.4895 | –202.2 | 0.6 |
| 10 | 3 | 8 | 9 | 3 | 7 | (11) | 16926.3226 | 87.3 | –5.1 |
| 10 | 3 | 8 | 9 | 3 | 7 | (12) | 16821.3130 | 176.5 | 1.1 |
| 11 | 0 | 11 | 10 | 0 | 10 | (00) | 15641.8743 | 144.3 | 9.1 |
| 11 | 0 | 11 | 10 | 0 | 10 | (10) | 15639.7832 | 42.0 | 36.2 |
| 11 | 0 | 11 | 10 | 0 | 10 | (01) | 15639.5961 | –0.2 | 16.9 |
| 11 | 0 | 11 | 10 | 0 | 10 | (12) | 15640.6415 | –124.8 | –1.2 |
| 11 | 0 | 11 | 10 | 1 | 10 | (10) | 15639.6469 | 8.3 | 2.6 |
| 11 | 0 | 11 | 10 | 1 | 10 | (01) | 15639.4681 | –17.9 | –0.8 |
| 11 | 1 | 10 | 10 | 1 | 9 | (00) | 16985.6298 | 147.9 | –0.7 |
| 11 | 1 | 10 | 10 | 1 | 9 | (10) | 16983.7582 | –1.1 | –1.2 |
| 11 | 1 | 10 | 10 | 1 | 9 | (01) | 16985.2860 | –114.3 | 0.6 |
| 11 | 1 | 10 | 10 | 2 | 9 | (00) | 16983.4086 | 151.5 | 0.7 |
| 11 | 1 | 10 | 10 | 2 | 9 | (10) | 16977.3468 | –12.4 | –1.8 |
| 11 | 1 | 10 | 10 | 2 | 9 | (01) | 16978.3490 | –114.0 | 1.3 |
| 11 | 1 | 10 | 10 | 2 | 9 | (12) | 16978.0016 | –16.7 | 1.6 |
| 11 | 1 | 11 | 10 | 0 | 10 | (00) | 15641.8745 | 135.5 | 0.2 |
| 11 | 1 | 11 | 10 | 0 | 10 | (10) | 15639.7704 | 1.8 | –4.1 |
| 11 | 1 | 11 | 10 | 0 | 10 | (01) | 15639.5961 | –29.5 | –12.3 |
| 11 | 1 | 11 | 10 | 0 | 10 | (11) | 15633.9545 | –37.8 | –33.4 |
| 11 | 1 | 11 | 10 | 0 | 10 | (12) | 15640.7745 | –124.4 | –0.7 |
| 11 | 1 | 11 | 10 | 1 | 10 | (00) | 15641.8479 | 143.3 | 8.1 |
| 11 | 1 | 11 | 10 | 1 | 10 | (01) | 15639.4808 | –34.5 | –17.3 |

| | | | | | | | | | |
|---|---|---|---|---|---|---|---|---|---|
| 11 | 2 | 9 | 10 | 2 | 8 | (10) | 18411.5562 | 91.0 | 0.4 |
| 11 | 2 | 9 | 10 | 2 | 8 | (01) | 18423.4214 | –196.0 | –1.2 |
| 11 | 2 | 9 | 10 | 3 | 8 | (10) | 18259.2424 | –161.6 | –2.0 |
| 11 | 2 | 9 | 10 | 3 | 8 | (01) | 18258.2974 | –204.8 | 3.0 |
| 11 | 2 | 9 | 10 | 3 | 8 | (12) | 18056.4335 | 228.7 | –0.9 |
| 11 | 2 | 10 | 10 | 1 | 9 | (00) | 16986.2920 | 148.0 | 0.2 |
| 11 | 2 | 10 | 10 | 1 | 9 | (10) | 16985.7060 | 2.7 | –1.1 |
| 11 | 2 | 10 | 10 | 1 | 9 | (01) | 16987.3718 | –114.5 | 0.6 |
| 11 | 2 | 10 | 10 | 2 | 9 | (00) | 16984.0706 | 151.4 | 1.4 |
| 11 | 2 | 10 | 10 | 2 | 9 | (10) | 16979.2951 | –8.1 | –1.2 |
| 11 | 2 | 10 | 10 | 2 | 9 | (01) | 16980.4346 | –114.4 | 1.1 |
| 11 | 3 | 8 | 10 | 3 | 7 | (01) | 20208.9720 | –225.7 | –0.1 |
| 11 | 3 | 9 | 10 | 2 | 8 | (00) | 18385.6185 | 118.5 | 0.4 |
| 11 | 3 | 9 | 10 | 2 | 8 | (10) | 18466.4541 | 197.8 | 2.5 |
| 11 | 3 | 9 | 10 | 2 | 8 | (01) | 18482.5357 | –201.7 | –4.2 |
| 11 | 3 | 9 | 10 | 3 | 8 | (10) | 18314.1403 | –54.8 | 0.0 |
| 11 | 3 | 9 | 10 | 3 | 8 | (01) | 18317.4124 | –209.8 | 0.7 |
| 12 | 0 | 12 | 11 | 0 | 11 | (00) | 17002.8905 | 142.7 | 4.2 |
| 12 | 0 | 12 | 11 | 0 | 11 | (10) | 17000.4503 | 5.5 | 2.4 |
| 12 | 0 | 12 | 11 | 0 | 11 | (01) | 17000.0176 | –35.5 | –11.6 |
| 12 | 0 | 12 | 11 | 0 | 11 | (11) | 16994.8627 | –6.7 | 6.1 |
| 12 | 0 | 12 | 11 | 1 | 11 | (00) | 17002.8763 | 137.6 | –0.9 |
| 12 | 0 | 12 | 11 | 1 | 11 | (10) | 17000.4163 | –1.0 | –4.1 |
| 12 | 0 | 12 | 11 | 1 | 11 | (01) | 16999.9995 | –24.3 | –0.5 |
| 12 | 0 | 12 | 11 | 1 | 11 | (11) | 16994.8511 | –10.7 | 2.0 |
| 12 | 1 | 11 | 11 | 2 | 10 | (00) | 18344.3985 | 151.6 | 1.3 |
| 12 | 1 | 11 | 11 | 2 | 10 | (10) | 18338.0573 | –5.4 | –1.2 |
| 12 | 1 | 11 | 11 | 2 | 10 | (01) | 18338.2491 | –127.3 | 1.3 |
| 12 | 1 | 12 | 11 | 0 | 11 | (00) | 17002.8906 | 140.4 | 1.9 |
| 12 | 1 | 12 | 11 | 0 | 11 | (10) | 17000.4503 | –1.7 | –4.9 |
| 12 | 1 | 12 | 11 | 0 | 11 | (01) | 17000.0176 | –43.1 | –19.3 |
| 12 | 1 | 12 | 11 | 0 | 11 | (11) | 16994.8511 | –20.2 | –7.5 |
| 12 | 1 | 12 | 11 | 0 | 11 | (12) | 17000.1372 | –143.7 | –0.6 |
| 12 | 1 | 12 | 11 | 1 | 11 | (00) | 17002.8906 | 149.5 | 11.0 |
| 12 | 1 | 12 | 11 | 1 | 11 | (01) | 16999.9995 | –32.0 | –8.1 |
| 12 | 2 | 10 | 11 | 2 | 9 | (10) | 19722.1757 | 37.9 | –3.1 |
| 12 | 2 | 10 | 11 | 3 | 9 | (10) | 19667.2819 | –64.8 | –1.1 |
| 12 | 2 | 10 | 11 | 3 | 9 | (01) | 19668.2279 | –224.1 | 1.2 |
| 12 | 2 | 11 | 11 | 1 | 10 | (00) | 18345.2508 | 149.8 | 0.5 |
| 12 | 2 | 11 | 11 | 1 | 10 | (10) | 18340.5796 | –1.0 | –1.6 |
| 12 | 2 | 11 | 11 | 1 | 10 | (01) | 18340.9448 | –127.0 | 1.8 |
| 12 | 2 | 11 | 11 | 2 | 10 | (01) | 18338.8534 | –132.5 | –3.8 |
| 12 | 3 | 10 | 11 | 2 | 9 | (10) | 19740.8551 | 83.1 | 1.5 |
| 12 | 3 | 10 | 11 | 3 | 9 | (10) | 19685.9562 | –24.8 | –1.6 |

| | | | | | | | | | |
|---|---|---|---|---|---|---|---|---|---|
| 13 | 0 | 13 | 12 | 0 | 12 | (00) | 18363.9344 | 140.4 | 0.5 |
| 13 | 0 | 13 | 12 | 1 | 12 | (00) | 18363.9344 | 142.7 | 2.9 |
| 13 | 0 | 13 | 12 | 1 | 12 | (10) | 18361.2636 | –5.7 | –4.8 |
| 13 | 0 | 13 | 12 | 1 | 12 | (01) | 18360.6523 | –33.8 | –1.3 |
| 13 | 0 | 13 | 12 | 1 | 12 | (11) | 18355.8040 | –17.8 | 4.7 |
| 13 | 1 | 12 | 12 | 2 | 11 | (00) | 19705.0057 | 150.8 | 2.5 |
| 13 | 1 | 12 | 12 | 2 | 11 | (10) | 19697.7876 | –2.5 | –1.5 |
| 13 | 1 | 12 | 12 | 2 | 11 | (01) | 19697.2868 | –142.0 | 2.0 |
| 13 | 1 | 13 | 12 | 0 | 12 | (00) | 18363.9344 | 139.7 | –0.1 |
| 13 | 1 | 13 | 12 | 0 | 12 | (10) | 18361.2738 | –4.7 | –3.7 |
| 13 | 1 | 13 | 12 | 0 | 12 | (01) | 18360.6638 | –31.9 | 0.6 |
| 13 | 1 | 13 | 12 | 0 | 12 | (11) | 18355.8040 | –20.3 | 2.2 |
| 13 | 1 | 13 | 12 | 1 | 12 | (00) | 18363.9340 | 141.7 | 1.9 |
| 13 | 2 | 11 | 12 | 3 | 10 | (10) | 21040.8166 | –21.3 | –1.2 |
| 13 | 2 | 11 | 12 | 3 | 10 | (01) | 21041.3548 | –249.0 | 1.2 |
| 13 | 2 | 12 | 12 | 1 | 11 | (00) | 19705.2503 | 148.9 | 0.9 |
| 13 | 2 | 12 | 12 | 1 | 11 | (10) | 19698.5289 | –0.6 | –1.2 |
| 13 | 2 | 12 | 12 | 1 | 11 | (01) | 19698.0700 | –142.4 | 1.8 |
| 13 | 3 | 11 | 12 | 2 | 10 | (01) | 21067.7225 | –257.1 | –1.0 |
| 14 | 0 | 14 | 13 | 0 | 13 | (00) | 19724.9968 | 140.0 | 0.9 |
| 14 | 0 | 14 | 13 | 1 | 13 | (00) | 19724.9968 | 140.6 | 1.5 |
| 14 | 0 | 14 | 13 | 1 | 13 | (10) | 19722.1752 | –10.3 | –3.7 |
| 14 | 0 | 14 | 13 | 1 | 13 | (01) | 19721.4076 | –42.0 | 1.3 |
| 14 | 0 | 14 | 13 | 1 | 13 | (11) | 19716.7945 | –29.5 | 4.4 |
| 14 | 0 | 14 | 13 | 1 | 13 | (12) | 19720.3135 | –189.3 | –1.7 |
| 14 | 1 | 13 | 13 | 2 | 12 | (10) | 21057.5359 | –0.4 | –1.0 |
| 14 | 1 | 13 | 13 | 2 | 12 | (01) | 21056.4861 | –154.7 | 6.9 |
| 14 | 1 | 14 | 13 | 0 | 13 | (00) | 19724.9968 | 139.8 | 0.7 |
| 14 | 1 | 14 | 13 | 0 | 13 | (10) | 19722.1752 | –12.7 | –6.1 |
| 14 | 1 | 14 | 13 | 0 | 13 | (01) | 19721.4076 | –44.5 | –1.2 |
| 14 | 1 | 14 | 13 | 0 | 13 | (11) | 19716.7945 | –30.1 | 3.7 |
| 14 | 1 | 14 | 13 | 0 | 13 | (12) | 19720.3135 | –201.1 | –13.5 |
| 14 | 1 | 14 | 13 | 1 | 13 | (00) | 19724.9968 | 140.4 | 1.3 |
| 14 | 2 | 13 | 13 | 1 | 12 | (10) | 21057.7487 | 0.0 | –1.1 |
| 14 | 2 | 13 | 13 | 1 | 12 | (01) | 21056.7042 | –159.4 | 2.2 |
| 15 | 0 | 15 | 14 | 0 | 14 | (00) | 21086.0687 | 138.2 | 2.0 |
| 15 | 0 | 15 | 14 | 1 | 14 | (00) | 21086.0685 | 138.2 | 2.0 |
| 15 | 0 | 15 | 14 | 1 | 14 | (10) | 21083.1326 | –18.4 | –4.4 |
| 15 | 0 | 15 | 14 | 1 | 14 | (01) | 21082.2342 | –55.4 | 1.0 |
| 15 | 0 | 15 | 14 | 1 | 14 | (11) | 21077.8163 | –41.5 | 5.6 |
| 15 | 0 | 15 | 14 | 1 | 14 | (12) | 21080.7843 | –211.1 | 1.8 |
| 15 | 1 | 15 | 14 | 0 | 14 | (00) | 21086.0685 | 138.0 | 1.8 |
| 15 | 1 | 15 | 14 | 0 | 14 | (10) | 21083.1326 | –19.0 | –5.0 |
| 15 | 1 | 15 | 14 | 0 | 14 | (01) | 21082.2342 | –56.0 | 0.3 |

| | | | | | | | | | |
|---|---|---|---|---|---|---|---|---|---|
| 15 | 1 | 15 | 14 | 0 | 14 | (11) | 21077.8163 | –41.7 | 5.4 |
| 15 | 1 | 15 | 14 | 0 | 14 | (12) | 21080.7843 | –214.2 | –1.3 |
| 15 | 1 | 15 | 14 | 1 | 14 | (00) | 21086.0685 | 138.2 | 2.0 |
| 16 | 0 | 16 | 15 | 1 | 15 | (10) | 22444.1252 | –28.1 | –4.9 |
| 16 | 0 | 16 | 15 | 1 | 15 | (01) | 22443.1153 | –71.3 | 0.5 |
| 16 | 0 | 16 | 15 | 1 | 15 | (11) | 22438.8587 | –56.6 | 5.8 |
| 16 | 1 | 16 | 15 | 0 | 15 | (00) | 22447.1450 | 133.2 | 2.3 |
| 16 | 1 | 16 | 15 | 0 | 15 | (10) | 22444.1252 | –28.3 | –5.0 |
| 16 | 1 | 16 | 15 | 0 | 15 | (01) | 22443.1153 | –71.4 | 0.4 |
| 16 | 1 | 16 | 15 | 0 | 15 | (11) | 22438.8587 | –56.7 | 5.8 |
| 16 | 1 | 16 | 15 | 1 | 15 | (00) | 22447.1450 | 133.2 | 2.3 |

**Table S6.** Molecular parameters of 35DMA in the principal axis system obtained using the *XIAM* program by fitting the rotational constants $F_{0,1}$ and the $F_{0,2}$ of the internal rotors.

| **Par.**[a] | **Unit** | **Fit *XIAM$_{mod}$*** | **Calc.**[b] |
|---|---|---|---|
| $A$ | MHz | 1737.266(30) | 1740.3 |
| $B$ | MHz | 1095.206(18) | 1092.6 |
| $C$ | MHz | 680.55809(65) | 679.7 |
| $V_{cc}$ | $cm^{-1}$ | –7.733(25) | –7.8 |
| $V_{3,1}$ | $cm^{-1}$ | 56.96(14) | 67.2 |
| $V_{3,2}$ | $cm^{-1}$ | 38.91(16) | 44.0 |
| $F_{0,1}$ | GHz | 152.42(39) | |
| $F_{0,2}$ | GHz | 164.43(64) | |
| $D_{\pi^2 J,1}$ | MHz | 2.36(28) | |
| $D_{\pi^2 J,2}$ | MHz | 0.0419(97) | |
| $V_{J,1}$ | MHz | –23.2(28) | |
| $V_{K,1}$ | MHz | –1.181(40) | |
| $V_{-,1}$ | MHz | 0.966(37) | |
| $V_{J,2}$ | MHz | –3.79(85) | |
| $V_{K,2}$ | MHz | 2.15(13) | |
| $V_{-,2}$ | MHz | –0.149(24) | |
| $\angle(i_1,a)$ | ° | 78.1177(17) | 78.6 |
| $\angle(i_1,b)$ | ° | 11.8823(17) | 11.4 |
| $\angle(i_1,c)$ | ° | 90.0[c] | 90.0 |
| $\angle(i_2,a)$ | | 41.8113(15) | 41.5 |
| $\angle(i_2,b)$ | | 131.8113(15) | 131.5 |
| $\angle(i_2,c)$ | | 90.0[c] | 90.0 |
| N[d] | | 622 | |
| *rms*[e] | kHz | 115.9 | |

[a] All molecular parameters refer to the principal axis system. Watson's S reduction I[r] representation were used. [b] Calculated at the B3LYP-D3BJ/6-311++G(d,p) level of theory. The rotational constants refer to the equilibrium structure. The centrifugal distortion constants are obtained from anharmonic frequency calculations. [c] Fixed due to symmetry. [d] Number of lines. [e] Root-means-square deviation of the fit.
